# Single‐nucleotide polymorphisms in the coding region of a disintegrin and metalloproteinase with thrombospondin motifs 4 and hepatocellular carcinoma: A retrospective case‐control study

**DOI:** 10.1002/cam4.2646

**Published:** 2019-10-30

**Authors:** Xing‐Zhizi Wang, Wei‐Zhong Tang, Qun‐Ying Su, Jin‐Guang Yao, Xiao‐Ying Huang, Qin‐Qin Long, Xue‐Min Wu, Qiang Xia, Xi‐Dai Long

**Affiliations:** ^1^ Department of Pathology The Affiliated Hospital of Youjiang Medical University for Nationalities Baise P.R. China; ^2^ Department of Gastrointestinal Surgery The Affiliated Tumor Hospital Guangxi Medical University Nanning P.R. China; ^3^ Department of Liver Surgery School of Medicine Ren Ji Hospital Shanghai Jiao Tong University Shanghai P.R. China

**Keywords:** *ADAMTS4*, HCC, prognosis, risk, SNPs

## Abstract

Previous studies have shown that single‐nucleotide polymorphisms (SNPs) of a disintegrin and metalloproteinase with thrombospondin type 1 motif 4 (*ADAMTS4*) may involve in the pathogenesis of some diseases. However, it is not clear whether they are associated with hepatocellular carcinoma (HCC). A hospital‐based case‐control study, including 862 cases with HCC and 1120 controls, was conducted to assess the effects of 258 SNPs in the coding regions of *ADAMTS4* on HCC risk and prognosis. We found that six SNPs in *ADAMTS4* were differential distribution between cases and controls via the primary screening analyses; however, only rs538321148 and rs1014509103 polymorphisms were further identified to modify the risk of HCC (odds ratio: 2.73 and 2.95; 95% confidence interval, 2.28‐3.29 and 2.43‐3.58; *P*‐value, 5.73 × 10^−27^ and 1.36 × 10^−27^, respectively). Significant interaction between these two SNPs and two known causes of hepatitis B virus and aflatoxin B1 were also observed. Furthermore, rs538321148 and rs1014509103 polymorphisms were associated not only with clinicopathological features of tumor such as tumor stage and grade, microvessel density, and vessel metastasis, but with poor overall survival. Additionally, these SNPs significantly downregulated *ADATMS4* expression in tumor tissues. These data suggest that SNPs in the coding region of *ADAMTS4*, such as rs538321148 and rs1014509103, may be potential biomarkers for predicting HCC risk and prognosis.

## Graphical abstract

This relatively large‐size retrospective study was conducted to investigate the association between ADAMTS4 single‐nucleotide polymorphisms and the risk and prognosis of hepatocellular carcinoma (HCC). Both rs538321148 and rs1014509103 polymorphisms were identified to increase HCC risk and change tumor outcome, and they may be potential biomarkers for predicting HCC risk and prognosis.

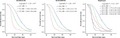

## 1 INTRODUCTION

Hepatocellular carcinoma (HCC), a major histological type of liver cancer, is the fifth most common malignant tumor worldwide and the third cause of cancer‐related mortality.^1^ More than half a million cases are diagnosed every year, there being major geographic differences in incidence. The annual incidence rates in eastern Asia, especially the People's Republic of China, exceed 15 per 100 000 inhabitants; whereas the figures are 5‐15 per 100 000 individuals in the southern Europe and Mediterranean basin and very low (less than 5/100 000) in northern Europe and America.^1^ It's worth noting that approximately 50% new‐diagnosed HCC cases locate in China. Evidence from clinical epidemiological studies has proved that chronic hepatitis B virus (HBV) infection and aflatoxin B1 (AFB1) is responsible for HCC carcinogenesis.^1^, ^2^ Additionally, growing evidence has also shown that genetic factors may play some kind of role in the control of hepatic tumorigenesis.^3^, ^4^, ^5^, ^6^ Results from high incident area of HCC display that genetic single nucleotide polymorphisms (SNPs) some genes (such as *XRCC1*, *ERCC2*, and so on) increase HCC risk and modify HCC prognosis^7^, ^8^; however, despite considerable advances in carcinogenetic fields, HCC‐related genes have been still elaborated less well.

A disintegrin and metalloproteinase with thrombospondin type 1 motif 4 (*ADAMTS4*) is an important member of a disintegrin and metalloproteinase with thrombospondin motifs (ADAMTS) family genes. This gene is located on chromosome 1q23.3 and consists of 9 exons (PubMed).^9^ Its encoding protein contains 837 amino acids and structurally includes following several domains: a signal peptide in N‐terminal (directing *ADAMTS4* to the secretory pathway), a pro‐domain (maintaining *ADAMTS4*'s enzyme latency), a metalloproteinase domain (binding zincs and having aggrecanase activity), a disintegrin‐like domain, a thrombospondin type 1 repeat domain, a cysteine‐rich domain, and a spacer region in C‐terminal.^10^, ^11^, ^12^ Functionally, *ADAMTS4* acts as an aggrecanase and involves in the degradation of aggrecan and brevican, which is responsible for collagen maturation, matrix degradation, organogenesis, and blood coagulation.^10^, ^13^, ^14^, ^15^ Recently, increasing evidence has indicated that the dysregulation of *ADAMTS4* may be associated with the pathogenesis of some tumors such as lung cancer,^16^ head and neck cancer,^17^ choriocarcinoma,^18^ Ewing's sarcoma,^19^ melanoma,^20^ colorectal cancer,^21^ breast cancer,^22^ HCC,^23^ and glioblastoma.^24^ Sever studies have reported that it also involves in the procession of angiogenesis and vascular endothelial growth factor receptor 2 (VEGFR2) phosphorylation.^25^ However, the relevance of the SNPs in *ADAMTS4* gene and HCC risk and prognosis remains to be elucidated. Here, we investigated whether 258 SNPs in the coding region of this gene are correlated with the risk and prognosis of HCC. Additionally, we also analyzed the potential effects of positive SNPs on *ADAMTS4* expression.

## 2 MATERIALS AND METHODS

### 2.1 Study population

A hospital‐based case‐control study was conducted in Guangxi Region, a high‐incidence area of HCC in China, according to our previously described criteria.^26^, ^27^, ^28^ Briefly, a total of 862 cases with histologically diagnosed HCC (representing 98% eligible cases) and 1120 age‐, gender‐, and race‐matched controls without any evidence of liver diseases (representing 97% eligible cases) were recruited in the affiliated hospitals of Youjiang Medical University for Nationalities and Guangxi Medical University during January 2009 and December 2013 and included in the present final analyses (Figure S1). For these individuals, 200 matched cases with HCC and controls were randomly selected for screening positive SNPs in *ADAMTS4* and the remainder was used for validation analyses. After signed consent was obtained, research samples (including 4 mL peripheral blood samples from every subjects and surgical removed tumor samples from every case with HCC) and corresponding clinicopathological data (including survival data) were collected.

In this study, individuals with positive hepatitis B surface antigen or anti‐hepatitis C virus (HCV) were considered as HBV or HCV infectors respectively. The exposure levels of AFB1 were evaluated using serum AFB1‐albumin adducts (se‐AAA) described in our previously published methods.^28^ Tumor stage and grade was defined according to WHO tumor, node, and metastasis system and Edmondson differentiation grading system; whereas liver cirrhosis and microvessel density (MVD) was evaluated using histopathological method.^29^ For survival analyses, all patients with HCC were followed and the follow‐up endpoint was set on 31 July 2018. Overall survival (OS) status was defined as the duration from the date of initial treatment to the date of death or last known date alive. The present study was approved by the ethic committees of the participating hospitals and was carried out in accordance with the approved guidelines (No. AYJM20090112). More detailed method information was available in the Supporting Information.

### 2.2 SNPs selection and genotypic analyses

Because epidemiological evidence has shown that the dysregulation of *ADAMTS4* may involve in HCC tumorigenesis^26^ and the SNPs in the coding region often result in the structural and functional alteration of the corresponding proteins, the SNPs resulting in missense mutations were obtained. According to the prediction value of Sorting Intolerant from Tolerant (SIFT, http://sift.bii.a-star.edu.sg) for SNPs in the coding region of *ADAMTS4* and the criteria of SIFT score less than 0.05, a total of 258 SNPs were selected for initial screening analyses in the first stage and six SNPs (rs150616368, rs538321148, rs17855812, rs1014509103, rs1485965919, and rs773407656) were further validated in the second stage. More detailed information was available in the Supporting Information.

### 2.3 *ADAMTS4* expression assays

The levels of *ADAMTS4* mRNA and protein were tested by TaqMan‐PCR and immunohistochemistry (IHC) techniques (described in the Supporting Information) respectively.

### 2.4 Statistical analysis

All statistical analyses were performed using the SPSS version 18 (SPSS Institute). The distribute difference between the groups was tested by chi‐square test, *t* test, or ANOVA test. To decrease false‐positive report probability in the multiple test,^30^ positive SNPs in *ADAMTS4* were screened and validated using non‐conditional regression based on the additive model according to the correlation matrix‐based method (CMBM).^31^ In brief, CMBM was used for correcting multiple test in the screening stage and accounted the linkage disequilibrium among *ADAMTS4* SNPs. Based on this method, *P*‐value < 1.94 × 10^−4^ was regarded as significant threshold for the main effects of SNPs in this study. Odds ratios (ORs) and corresponding 95% confidence intervals (CIs) were calculated using multivariate conditional logistic regression model (including known causes of Guangxiese HCC). The effects of *ADAMTS4* SNPs on the OS of HCC were elucidated by Kaplan‐Meier survival model and the Cox multivariate regression model. Spearman's correlation analyses were used to test the association between ADATMS4 SNPs and the amount of *ADAMTS4* protein. More detailed statistical analyses information was available in the Supporting Information.

## 3 RESULTS

### 3.1 The characteristics of subjects

Table S1 summarized the baseline characteristics of all subjects. Demographic features (including gender, age, race, smoking and drinking status, and HCV infection information) between cases with HCC and controls were not significantly different. However, cases with HCC featured higher HBV infection (OR = 7.88) and AFB1 exposure (OR = 3.52). Results from joint analyses further showed that individuals with both positive HBV status and AFB1 exposure faced a higher risk of HCC than those without risk factors or with one risk factor (OR 23.64; 95% CI 17.15‐32.58) (Table S2).

### 3.2 *ADAMTS4* SNPs and HCC risk

Genotypic distribution of all selected *ADAMTS4* SNPs among control individuals was the Hardy‐Weinberg equilibrium. Results in the screening stage showed that six SNPs (rs150616368, rs538321148, rs17855812, rs1014509103, rs1485965919, and rs773407656) were significantly associated with HCC risk (Table S3); however, only two of them (rs538321148 and rs1014509103) were further identified as risk factors for HCC via the validation analyses (Table 1). Results from the combination of all individuals (Merged set) exhibited that the rs538321148 and rs1014509103 polymorphisms significantly increased HCC risk [ORs (95% CIs), 2.73 (2.28‐3.29) and 2.95 (2.43‐3.58) respectively] (Table 1). In the risk analyses of *ADAMTS4* haplotypes on the basis of these two SNPs, the risk value for HCC would gradually increase with the increment of haplotypes with risk alleles (ORs 1.46‐39.87). Totally, these individuals having risk‐allele haplotypes of rs538321148 or rs1014509103 (Haplotype‐P), compared to those without risk‐allele haplotypes (Haplotype‐N), featured higher HCC risk (OR 2.45; 95% CI 2.04‐2.95) (Table 2).

**Table 1:** ADAMTS4 SNPs and HCC risk

No.	ADAMTS4	Screening set		Validation set		Merged set	
		*N*_con/hcc_ a	OR (95% CI/*P*_trend_)^b^	*N*_con/hcc_	OR (95% CI/*P*_trend_)	*N*_con/hcc_	OR (95% CI/*P*_trend_)
SNP008	Rs150616368‐GG	153/108	Reference	648/448	Reference	801/556	Reference
	GA	41/52	1.80 (1.12‐2.90/0.02)	212/172	1.17 (0.93‐1.48/0.18)	253/224	1.28 (1.04‐1.58/0.02)
	AA	6/40	9.44 (3.86‐23.06/8.48 × 10^−7^)	60/42	1.01 (0.67‐1.52/0.97)	66/82	1.79 (1.27‐2.51/8.86 × 10^−4^)
	GA/AA	47/92	2.78 (1.81‐4.27/3.25 × 10^−6^)	272/214	1.14 (0.92‐1.41/0.24)	319/306	1.38 (1.14‐1.67/8.72 × 10^−4^)
SNP021	Rs538321148‐CC	135/84	Reference	607/276	Reference	742/360	Reference
	CT	62/90	2.36 (1.54‐3.61/7.40 × 10^−5^)	274/298	2.39 (1.92‐2.97/7.89 × 10^−15^)	336/388	2.38 (1.96‐2.89/1.23 × 10^−18^)
	TT	3/26	14.02 (4.11‐47.78/2.45 × 10^−5^)	39/88	4.99 (3.33‐7.49/6.95 × 10^−15^)	42/114	5.59 (3.84‐8.14/2.66 × 10^−19^)
	CT/TT	65/116	2.88 (1.91‐4.33/3.97 × 10^−7^)	313/386	2.71 (2.20‐3.34/5.63 × 10^−21^)	378/502	2.73 (2.28‐3.29/5.73 × 10^−27^)
SNP105	Rs17855812‐TT	174/138	Reference	771/542	Reference	945/680	Reference
	TC	24//57	3.04 (1.79‐5.16/3.70 × 10^−5^)	126/110	1.24 (0.94‐1.64/0.12)	150/167	1.55 (1.22‐1.97/3.90 × 10^−4^)
	CC	2/5	2.90 (0.55‐15.30/0.21)	23/10	0.61 (0.29‐1.29/0.19)	25/15	0.83 (0.43‐1.58/0.56)
	TC/CC	26/62	3.03 (1.82‐5.05/2.10 × 10^−5^)	149/120	1.15 (0.88‐1.49/0.32)	175/182	1.45 (1.15‐1.82/1.70 × 10^−3^)
SNP141	Rs1014509103‐GG	155/112	Reference	717/357	Reference	872/469	Reference
	GA	37/54	2.02 (1.24‐3.27/4.52 × 10^−3^)	167/181	2.20 (1.72‐2.82/3.99 × 10^−10^)	204/235	2.14 (1.72‐2.67/8.39 × 10^−12^)
	AA	8/34	5.87 (2.62‐13.17/1.75 × 10^−5^)	36/124	6.92 (4.67‐10.27/6.29 × 10^−22^)	44/158	6.68 (4.69‐9.50/4.75 × 10^−26^)
	GA/AA	45/88	2.67 (1.74‐4.15/8.02 × 10^−6^)	203/305	3.04 (2.44‐3.79/3.84 × 10^−23^)	248/393	2.95 (2.43‐3.58/1.36 × 10^−27^)
SNP152	Rs1485965919‐CC	170/133	Reference	767/539	Reference	937/672	Reference
	CA	26/59	2.98 (1.73‐4.85/5.31 × 10^−5^)	136/108	1.11 (0.91‐1.36/0.30)	162/167	1.44 (1.14‐1.83/2.67 × 10^−3^)
	AA	4/8	2.54 (0.75‐8.65/0.14)	17/15	1.13 (0.90‐1.41/0.29)	21/23	1.54 (0.85‐2.81/0.16)
	CA/AA	30/67	2.85 (1.75‐4.64/2.68 × 10^−5^)	153/123	1.15 (0.88‐1.49/0.31)	183/190	1.45 (1.16‐1.82/1.24 × 10^−3^)
SNP171	Rs773407656‐GG	186/158	Reference	858/615	Reference	1044/773	Reference
	GA	12/34	3.32 (1.66‐6.34/6.69 × 10^−4^)	53/27	0.71 (0.44‐1.14/0.16)	65/61	1.27 (0.88‐1.82/0.20)
	AA	2//8	4.80 (1.00‐22.99/0.05)	9/20	3.08 (1.40‐6.83/5.37 × 10^−3^)	11/28	3.42 (1.69‐6.92/6.11 × 10^−4^)
	GA/AA	14/42	3.53 (1.86‐6.71/1.14 × 10^−4^)	62/47	1.05 (0.71‐1.56/0.790	76/89	1.58 (1.15‐2.18/5.12 × 10^−3^)

Abbreviations: HCC, hepatocellular carcinoma; OR, odds ratio; SNP, single‐nucleotide polymorphism.

a *N*_con/hcc_ refers to the number of controls/the number of cases with hepatocellular carcinoma.

b OR conditional on matched set.

**Table 2:** The haplotypes of ADAMTS4 rs538321148 and rs1014509103 polymorphisms and HCC risk

Haplotypes^a^	Controls, n (%)	HCCs, n (%)	OR (95% CI)^b^	*P*_trend_
Haplotype‐N	608 (54.3)	281 (32.6)	Reference	
Haplotype‐1	352 (31.4)	237 (27.5)	1.46 (1.17‐1.81)	6.96 × 10^−4^
Haplotype‐2	124 (11.1)	176 (20.4)	3.07 (2.34‐4.02)	4.09 × 10^−16^
Haplotype‐3	32 (2.9)	94 (10.9)	6.39 (4.17‐9.78)	1.36 × 10^−17^
Haplotype‐4	4 (0.4)	74 (8.6)	39.87 (14.43‐110.14)	1.16 × 10^−12^
Haplotype‐P	512 (45.7)	581 (67.4)	2.45 (2.04‐2.95)	1.62 × 10^−21^

Abbreviations: HCC, hepatocellular carcinoma; OR, odds ratio.

a Haplotype‐P and ‐N refers to ADAMTS4 haplotype with or without risk alleles (OR > 1) of rs538321148 and rs1014509103, respectively; whereas haplotype‐1 to 4 represents ADAMTS4 haplotype with 1 to 4 risk alleles of rs538321148 and rs1014509103, respectively.

b OR conditional on matched set.

### 3.3 Joint effects of *ADAMTS4* SNPs and causes on HCC risk

Given that aforementioned HBV infection and AFB1 exposure are two major causes for Guangxiese HCC,^2^ we questioned whether there were interactive effects between causes and *ADAMTS4* SNPs and accomplished a series of joint analyses based on the combination of *ADAMTS4* genotypes and causes (Table 3). Results showed that these individuals with both positive status of causes and risk genotypes would face higher risk for HCC. For example, positive status of HBV infection significantly increased HCC risk (OR = 6.16 for rs538321148‐CC and 6.74 for rs1014509103‐GG, respectively); this risk value was more pronounced among individuals with ADATMS4 risk genotypes (corresponding OR, 19.97 for rs538321148‐CT/TT and 19.45 for rs1014509103‐GA/AA respectively). According to the environment‐gene interaction formula [OReg > (OReg' × ORe'g)],^32^ there was evidence of multiplicatively interactive effects of causes and *ADAMTS4* genotypes on HCC risk [ex., 6.90 > (1.76 × 1.03) for AFB1 interacting with *ADAMTS4* haplotypes]. To further identify their interactive value, we finished multiplicatively interactive analyses using multivariable logistic regression model (including all potential interactive variables) on the basis of stepwise forward selection with a likelihood ratio test (Table S4). Results proved significantly multiplicative interaction between *ADAMTS4* SNPs and causes (including HBV and AFB1 status).

**Table 3:** Joint effects of HBV infective status and adamts4 SNPs on HCC risk

Variables	Controls, n (%)	HCCs, n (%)	OR (95% CI)^a^	*P*_trend_
HBV (−)				
Rs538321148‐CC	536 (47.9)	107 (12.4)	Reference	
CT	241 (21.5)	86 (10.0)	1.79 (1.30‐2.47)	4.03 × 10^−4^
TT	39 (3.5)	26 (3.0)	3.35 (1.95‐5.76)	1.16 × 10^−5^
CT/TT	280 (25.0)	112 (13.0)	2.06 (1.48‐2.71)	6.33 × 10^−6^
HBV (+)				
Rs538321148‐CC	206 (18.4)	253 (29.4)	6.16 (4.66‐8.12)	9.84 × 10^−38^
CT	95 (8.5)	302 (35.0)	15.95 (11.69‐21.78)	4.52 × 10^−68^
TT	3 (0.3)	88 (10.2)	146.96 (45.65‐473.17)	6.03 × 10^−17^
CT/TT	98 (8.8)	390 (45.2)	19.97 (14.73‐27.07)	7.47 × 10^−83^
HBV (−)				
Rs1014509103‐GG	643 (57.4)	138 (16.0)	Reference	
GA	134 (12.0)	45 (5.2)	1.56 (1.07‐2.30)	0.02
AA	39 (3.5)	36 (4.2)	4.30 (2.64‐7.02)	5.01 × 10^−9^
GA/AA	173 (15.4)	81 (9.4)	2.19 (1.58‐3.02)	1.93 × 10^−6^
HBV (+)				
Rs1014509103‐GG	229 (20.4)	331 (38.4)	6.74 (5.25‐8.64)	8.74 × 10^−51^
GA	70 (6.3)	190 (22.0)	12.65 (9.08‐17.63)	7.42 × 10^−51^
AA	5 (0.4)	122 (14.2)	113.71 (45.63‐283.35)	2.96 × 10^−24^
GA/AA	75 (6.7)	312 (36.2)	19.45 (14.22‐26.60)	5.20 × 10^−77^
HBV (−)				
Haplotype‐N^b^	442 (39.5)	83 (9.6)	Reference	
P^b^	374 (33.4)	136 (15.8)	1.93 (1.42‐2.63)	2.52 × 10^−5^
HBV (+)				
N^b^	166 (14.8)	198 (23.0)	6.36 (4.65‐8.68)	3.93 × 10^−31^
P^b^	138 (12.3)	445 (51.6)	17.27 (12.76‐23.38)	7.06 × 10^−76^
AFB1 (−)				
Rs538321148‐CC	357 (31.9)	114 (13.2)	Reference	
CT	207 (18.5)	68 (7.9)	1.03 (0.73‐1.45)	0.87
TT	22 (2.0)	14 (1.6)	1.99 (0.99‐4.02)	0.06
CT/TT	229 (20.4)	82 (9.5)	1.12 (0.81‐1.56)	0.49
AFB1 (+)				
Rs538321148‐CC	385 (34.4)	246 (28.5)	2.01 (1.54‐2.62)	2.44 × 10^−7^
CT	129 (11.5)	320 (37.1)	7.80 (5.81‐10.46)	1.08 × 10^−42^
TT	20 (1.8)	100 (11.6)	15.70 (9.29‐26.53)	7.85 × 10^−25^
CT/TT	149 (13.3)	420 (48.7)	8.86 (6.68‐11.74)	6.31 × 10^−52^
AFB1 (−)				
Rs1014509103‐GG	440 (39.3)	127 (14.7)	Reference	
GA	108 (9.6)	45 (5.2)	1.45 (0.97‐2.16)	0.07
AA	38 (3.4)	24 (2.8)	2.20 (1.27‐3.81)	4.74 × 10^−3^
GA/AA	146 (13.0)	69 (8.0)	1.65 (1.16‐2.33)	5.05 × 10^−3^
AFB1 (+)				
Rs1014509103‐GG	432 (38.6)	342 (39.7)	2.76 (2.16‐3.52)	3.22 × 10^−16^
GA	96 (8.6)	190 (22.0)	6.88 (5.02‐9.43)	3.80 × 10^−33^
AA	6 (0.5)	134 (15.5)	77.48 (33.40‐179.73)	3.96 × 10^−24^
GA/AA	102 (9.1)	324 (37.6)	11.04 (8.20‐14.87)	2.57 × 10^−56^
AFB1 (−)				
Haplotype‐N^b^	285 (25.4)	94 (10.9)	Reference	
P^b^	301 (26.9)	102 (11.8)	1.03 (0.74‐1.42)	0.87
AFB1 (+)				
Haplotype‐N^b^	323 (28.8)	187 (21.7)	1.76 (1.31‐2.37)	1.62 × 10^−4^
P^b^	211 (18.8)	479 (55.6)	6.90 (5.20‐9.17)	1.52 × 10^−40^

Abbreviation: AFB1, aflatoxin B1; CI, confidence interval; HBV, hepatitis B virus; HCC, hepatocellular carcinoma; OR, odds ratio.

a OR conditional on matched set.

b Haplotype‐P and ‐N refers to ADAMTS4 haplotype with or without risk alleles of rs538321148 and rs1014509103, respectively.

### 3.4 The effects of *ADAMTS4* SNPs on OS of patients with HCC

**Figure 1:**
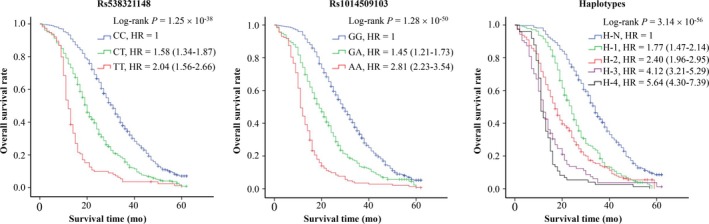
ADAMTS4 rs538321148 and rs1014509103 polymorphisms significantly correlating with hepatocellular carcinoma prognosis. Cumulative hazard function was plotted by Kaplan‐Meier's methodology, and *P* value was calculated with two‐sided log‐rank tests. The relative hazard ratio values for genotypes were calculated using multivariable cox regression models (with all significant variables) based on forward‐step method with likelihood ratio test. Abbreviations: H‐1, ADAMTS4 haplotype with 1 risk allele of rs538321148 and rs1014509103; H‐2, ADAMTS4 haplotype with 2 risk alleles of rs538321148 and rs1014509103; H‐3, ADAMTS4 haplotype with 3 risk alleles of rs538321148 and rs1014509103; H‐4, ADAMTS4 haplotype with 4 risk alleles of rs538321148 and rs1014509103; H‐N, ADAMTS4 haplotype without risk alleles of rs538321148 and rs1014509103; HR, hazard ratio

To explore the effects of *ADAMTS4* SNPs on OS of patients with HCC, we first analyzed the association between *ADAMTS4* rs538321148 and rs1014509103 polymorphisms and clinicopathological features of HCC using non‐conditional regression models (Table 4). We found that these two SNPs and their haplotypes were significantly correlated with increasing se‐AAA level, larger tumor size, tumor dedifferentiation, higher tumor, node and metastasis staging system stage, increasing microvessel density and portal vein tumor thrombus risk, but not with other features. Next, we investigated the relationship between *ADAMTS4* SNPs and OS of HCC cases and observed that *ADAMTS4* rs538321148 and rs1014509103 polymorphisms and their haplotypes not only shortened OS time but also increased the death risk of patients with HCC [hazard ratio (95% CI), 2.04 (1.56‐2.66) for rs538321148‐TT, 2.81 (2.23‐3.54) for rs1014509103‐AA, and 5.64 (4.30‐7.39) for Haplotype with both rs538321148‐TT and rs1014509103‐AA respectively] (Figure 1). Taken together, these results are indicative of *ADAMTS4* SNPs as potential biomarkers for HCC prognosis.

**Table 4:** The ADAMTS4 SNPs and the clinic‐pathological features of HCC

	Rs538321148		Rs1014509103		Haplotypes of ADAMTS4	
	n^a^	OR (95% CI/*P*_trend_)	n^b^	OR (95% CI/*P*_trend_)	n^c^	OR (95% CI/*P*_trend_)
Total	360/502	/	469/393	/	281/581	/
Gender						
Female	102/131	Reference	119/114	Reference	73/160	Reference
Male	258/371	0.94 (0.68‐1.28/0.68)	350/279	0.83 (0.62‐1.12/0.3)	208/421	0.73 (0.42‐1.25/0.25)
Age (y)^d^						
≤49	192/250	Reference	250/192	Reference	155/287	Reference
>49	168/252	1.09 (0.82‐1.44/0.56)	219/201	1.19 (0.91‐1.56/0.20)	126/294	1.08 (0.68‐1.69/0.76)
Ethnicity						
Han	181/233	Reference	228/186	Reference	146/268	Reference
Zhuang	179/269	1.17 (0.88‐1.56/0.27)	241/207	1.12 (0.81‐1.54/0.50)	135/313	1.16 (0.86‐1.58/0.34)
Smoking						
No	267/378	Reference	344/301	Reference	200/445	Reference
Yes	93/124	1.06 (0.81‐1.46/0.72)	125/92	0.84 (0.62‐1.15/0.27)	81/136	0.81 (0.49‐1.33/0.40)
Drinking						
No	265/379	Reference	354/290	Reference	209/435	Reference
Yes	95/123	0.90 (0.66‐1.23/0.51)	115/103	1.09 (0.80‐1.49/0.58)	72/146	1.08 (0.81‐1.43/0.61)
HBV infection						
Negative	107/112	Reference	138/81	Reference	83/136	Reference
Positive	253/390	2.41 (0.93‐6.19/0.07)	331/312	1.90 (0.79‐4.61/0.15)	198/445	1.59 (0.60‐4.25/0.36)
HCV infection status						
Negative	296/407	Reference	382/321	Reference	229/474	Reference
Positive	64/95	0.93 (0.54‐1.61/0.79)	87/72	0.89 (0.47‐1.69/0.72)	52/107	0.95 (0.50‐1.80/0.88)
AFB1 exposure status						
Negative	114/82	Reference	127/69	Reference	94/102	Reference
Positive	246/420	1.96 (1.39‐2.77/1.37 × 10^−4^)	342/324	1.81 (1.08‐2.00/0.02)	187/479	1.88 (1.32‐2.67/5.20 × 10^−4^)
Tumor size						
≤5 cm	60/55	Reference	79/36	Reference	48/67	Reference
>5 cm	300/447	1.64 (1.10‐2.43/0.01)	390/357	2.02 (1.32‐3.07/1.07 × 10^−3^)	233/514	1.60 (1.07‐2.39/0.02)
Tumor grade^e^						
Low	197/175	Reference	241/131	Reference	167/205	Reference
High	163/327	1.82 (1.35‐2.45/9.13 × 10^−5^)	228/262	1.64 (1.23‐2.23/1.03 × 10^−3^)	114/376	2.06 (1.50‐2.83/8.47 × 10^−6^)
TNM stage						
I	43/29	Reference	59/13	Reference	38/34	Reference
II	241/285	1.31 (0.58‐2.95/0.52)	314/212	3.13 (1.30‐7.53/0.01)	196/330	1.94 (0.79‐4.72/0.15)
III	76/188	2.44 (1.04‐5.72/0.04)	96/168	7.56 (3.02‐18.88/1.50 × 10^−5^)	47/217	4.73 (1.83‐12.23/1.35 × 10^−3^)
MVD^f^						
Low	240/169	Reference	288/121	Reference	199/210	Reference
High	120/333	3.93 (2.95‐5.23/1.02 × 10^−20^)	181/272	3.58 (2.69‐4.75/1.36 × 10^−18^)	82/371	4.26 (3.13‐5.80/3.09 × 10^−20^)
PVT						
No	124/88	Reference	146/66	Reference	108/104	Reference
Yes	236/414	1.82 (1.29‐2.56/5.85 × 10^−4^)	323/327	1.68 (1.17‐2.40/4.50 × 10^−3^)	173/477	2.01 (1.42‐2.85/8.85 × 10^−5^)
Liver cirrhosis						
No	114/128	Reference	149/93	Reference	90/152	Reference
Yes	246/374	0.63 (0.25‐1.59/0.33)	320/300	0.91 (0.39‐1.61/0.83)	191/429	1.30 (0.95‐1.78/0.10)

Abbreviations: AFB1, aflatoxin B1; CI, confidence interval; MVD, microvessel density; OR, odds ratio.

a The number of cases with rs538321148‐CC genotype/the number of cases with rs538321148‐CT/TT genotypes.

b The number of cases with rs1014509103‐GG genotype/the number of cases with rs1014509103‐GA/AA genotype genotypes.

c The number of cases with ADAMTS4 Haplotype‐N/the number of cases with ADAMTS4 Haplotype‐P.

d The age of cases was classed into two groups according to the mean age of cases.

e Tumor grade was classed into two groups, low grade (Edmondson grade I and II) and high grade (Edmondson grade III and IV), according to Edmondson differentiation grading system.

f MVD was classed into two groups, low grade (≤50/×200 magnifications) and high grade (>50/×200 magnifications), according to the number of MVD in the tumor tissues.

### 3.5 The effects of *ADAMTS4* SNPs on the expression of *ADAMTS4*

**Figure 2:**
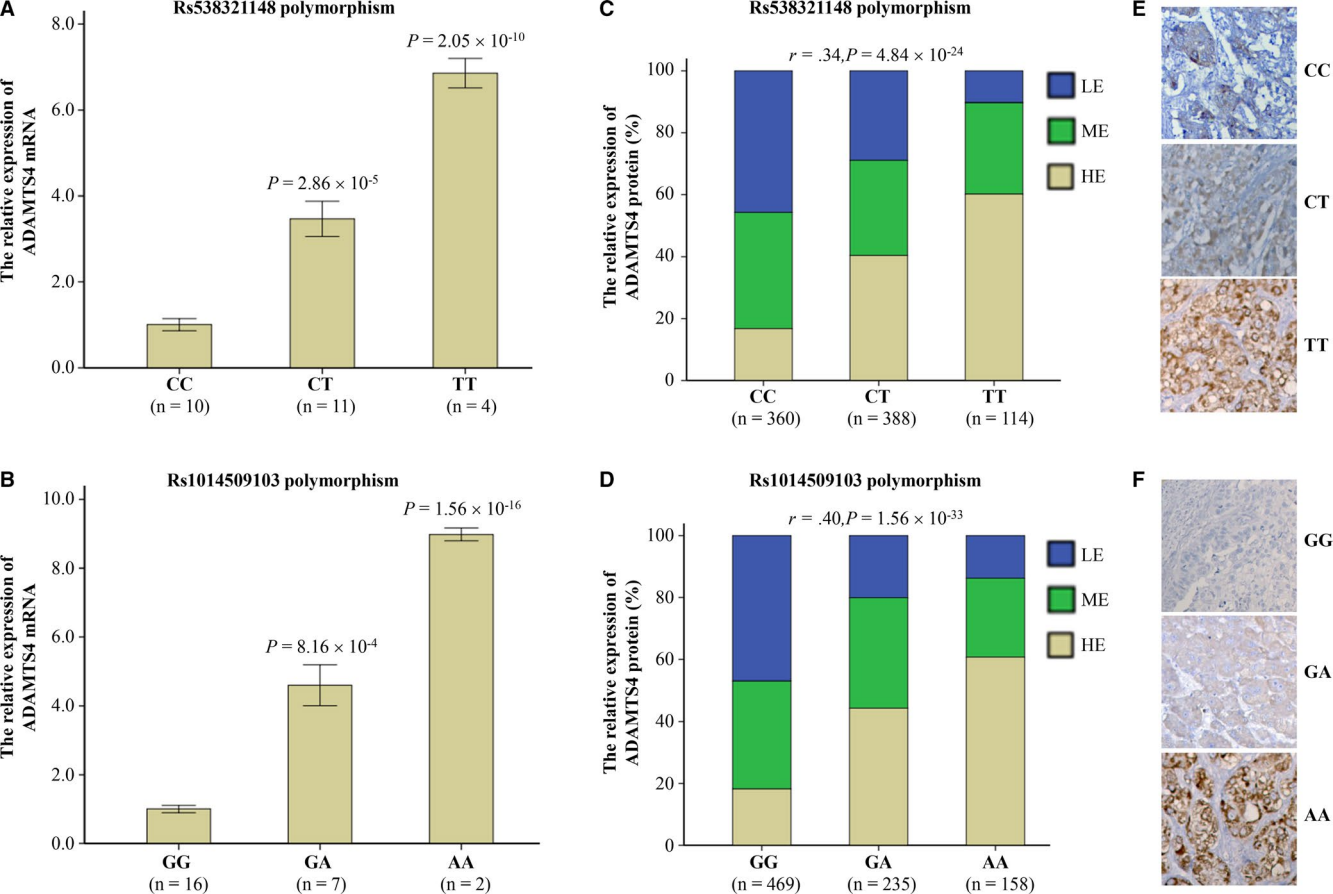
ADAMTS4 rs538321148 and rs1014509103 polymorphisms significantly associating with ADAMTS4 expression. A and B, The ADAMTS4 mRNA levels in the fresh tissues with hepatocellular carcinoma (n = 25) were tested using TaqMan real‐time quantitative PCR method and its relative expression was calculated by the comparative CT method. Data were shown as means ± SD and tested by ANOVA test. C‐F, The protein amount of ADAMTS4 in the paraffin‐embedded tissues with HCC (n = 862) were tested using immunohistochemistry method. To analyze, the levels of ADAMTS4 protein were divided into three groups: low expression group (LE, immunoreaction score ≤ 4), medium expression group (ME, 4 < IRS < 8), and high expression group (HE, IRS ≥ 8). The associations between the levels of ADAMTS4 protein and either rs538321148 (C) or rs1014509103 (D) were analyzed using Spearman's correlation model. Represent immunohistochemistry plots shows their associations (E, F)

In view of previous studies showing that the dysregulation of *ADAMTS4* expression may involve in carcinogenesis,^16^, ^18^, ^21^, ^26^ we questioned whether *ADAMTS4* SNPs altered the expression of *ADAMTS4* mRNA and protein. To answer it, we collected 25 fresh tissue samples with HCC and tested the amount of *ADAMTS4* mRNA. Results from real‐time quantitative PCR displayed that both rs538321148 and rs1014509103 polymorphisms were correlated with increasing *ADAMTS4* mRNA expression (Figure 2A,B). Immunohistochemistry data further proved that these two SNPs were positively associated with the levels of *ADAMTS4* protein (*r* = .34 for rs538321148 and 0.40 for rs1014509103, respectively) (Figure 2C,D). Represent IHC plots showed their relationship (Figure 2E,F).

## 4 DISCUSSION

Through a hospital‐based case‐control study, we explored the linkage between *ADAMTS4* SNPs and the risk of HCC in Guangxi Zhuang Autonomous Region, a high incident area of liver cancer in China. It was found that *ADAMTS4* genotypes with rs538321148 T alleles and rs1014509103 A alleles and their haplotypes had a substantial correlation with the growing risk of HCC (ORs, 2.73 for rs538321148, 2.95 for rs1014509103, and 6.11 for their haplotypes respectively). Interestingly, the interactions between *ADAMTS4* genotypes and environmental factors HBV and AFB1 were also found for HCC risk. These results may indicate that the potential of *ADAMTS4* SNPs as biomarkers for the risk of HCC.

In Guangxi area, HCC is frequent and one of the most common malignant types; the most important causes of which are AFB1 exposure and HBV infection.^2^ Our previous reports and prevent study also show that patients with HCC had a higher frequency of positive AFB1 exposure and HBV infection 3, 33; furthermore, we observed the significant evidence of multiplicatively interactive effects of AFB1 and HBV on HCC risk. However, the fact that among these individuals exposing to the same risk factors, only small proportion develop cancer is indicative of individual's genetic susceptibility to HCC carcinogenesis.^3^, ^4^, ^5^, ^6^ Therefore, the new approach to understand genetic susceptible factors during triggering the tumorigenesis of hepatic cells is of high priority in current perspective.

Human *ADAMTS4* (also known as *ADMP‐1*, *ADAMTS‐2*, or *ADAMTS‐4*), a major member of ADAMTS family genes, is an important aggrecanase involving in collagen maturation, matrix degradation, organogenesis, and blood coagulation.^10^, ^14^ In the past several decades, the epidemiological and clinicopathological studies have shown that the dysregulation of *ADAMTS4* expression and function may involve in the process of some tumors, including head and neck cancer, melanoma, choriocarcinoma, and Ewing's sarcoma.^17^, ^18^, ^19^, ^20^ Demircan et al^17^ investigated the expression of *ADAMTS4* mRNA in primary head and neck squamous cell carcinoma with and without metastasis by quantitative real time polymerase chain reaction technique and found that higher expression was in the metastatic tumor tissues. Lee^18^ and Minobe^19^ observed that the different expression of *ADAMTS4* involves in the process of choriocarcinoma and Ewing's sarcoma. Rao et al^20^ further analyzed differential functions of different catalytic fragments of *ADAMTS4* in the melanoma growth and angiogenesis. They observed that both *ADAMTS4* full‐length and N‐terminal autocatalytic fragment (NTAF) can promote angiogenesis and tumor growth in mice; whereas their mutant types or C‐terminal fragment displayed converse role. Of particular concerns are the coexistence of proteolytic fragments which contain only C‐terminal ancillary domains), the full‐length fragments, and NTAFs in the culture cells and tissues samples with melanoma.^20^ Our previous report^26^ and the present study shows that the dysregulation of *ADAMTS4* by genetic factors such as mutant microRNA‐1268a increase the risk of HCC. Together, the collected data imply that *ADAMTS4* may play some vital roles in the procession of carcinogenesis, and thus deserve to be further evaluated.

More than 3200 SNPs in the *ADAMTS4* have been identified and the part of them may involve in human diseases such as lumbar disc degeneratio.^34^, ^35^, ^36^ For example, Liu et al^34^ investigated the association between five SNPs (rs11585858, rs4656291, rs41270041, rs10908826 and rs4233367) in and around region of ADAMTS4 via a case‐control study (including 482 cases with lumber intervertebral disc degeneration and 496 controls without any evidence of diseases) and observed that only rs4233367 altered the risk of lumber intervertebral disc degeneration (OR 0.69; 95% CI 0.50‐0.94; *P* = 1.66 × 10^−2^). However, Canbek et al^36^ reported that two SNPs (rs226794 and rs2830585) in the ADAMTS4 did not change knee osteoarthritis risk. Until now, there have been any meta‐analyses focused on this topic because of only two known studies.^35^ In this study, 258 known SNPs in the coding regions of *ADAMTS4* gene were selected initially for the first‐stage screening analyses, mainly because of their implied effects on the structure and function of *ADAMTS4* protein by SIFT method (http://sift.bii.a-star.edu.sg). Of these SNPs analyzed, only rs538321148 (Val^104^ to Iso^104^) and rs1014509103 (Thr^493^ to Iso^493^) polymorphism were found to link with increasing HCC risk through the screening‐ and validation‐stage analyses. Moreover, they were also observed to significantly interact with HBV infection and AFB1 exposure during HCC carcinogenesis. These data indicate that rs538321148 and rs1014509103 polymorphisms may act as potential susceptibility factors for cancer risk, and the possibility of the genetic SNPs in *ADAMTS4* influencing HCC risk should therefore not be ignored.

To explore possible pathogenesis of *ADAMTS4* SNPs modifying HCC risk, we examined the expression of *ADAMTS4* mRNA and protein and the pathological characteristics of HCC. Our results showed that *ADAMTS4* rs538321148 and rs1014509103 polymorphisms were linked with the upregulation of *ADAMTS4* expression, increasing tumor MVD and metastasis risk, and tumor dedifferentiation and progression. Several recent in vitro and in vivo studies^20^, ^23^, ^25^ and clinicopathological investigations^18^, ^19^, ^23^, ^25^ have exhibited that the dysregulation of *ADAMTS4* expression promotes tumor progression and angiogenesis by modifying pVEGFR2. The OS analyses further proved that these two SNPs were significantly relevant to poor survival of patients with HCC, emphasizing the potential values of *ADAMTS4* rs538321148 and rs1014509103 polymorphisms in predicting HCC prognosis. Collectively, these findings imply that *ADAMTS4* SNPs could affect the expression and function of *ADAMTS4* and ultimately increase HCC risk and shorten the prognosis of patients with this cancer.

The major strongpoints of this study were the retrospective two‐stage design, relatively large‐size samples, and SNPs screening strategy on the basis of SIFT method. The potential effects of known confounders (including gender, age, and race) for Guangxiese HCC were controlled using an individually matched case‐control design. In addition, the most two causes of HCC among Guangxi population, also HBV infection and AFB1, were included in the current study and their interaction with SNPs in the *ADAMTS4* were also elucidated for HCC risk.

However, there were several disadvantages to limit our study. First, the hospital‐based design may result in the selective bias of study subjects. Second, the gene‐environmental interactions may be underestimated because of the relatively small sample sizes of some subgroups and low frequency of risk alleles and genotypes, especially some *ADAMTS4* haplotypes. Third, although SNPs in the coding region of *ADAMTS4* were analyzed in this study, SNPs in the 5′ near region, 5′‐UTR, 3′‐UTR, and 3′ near region of this gene may change the expression of *ADAMTS4* and affect HCC carcinogenesis. Fourth, some SNPs in the coding region of *ADAMTS4* (such as rs377253620, rs1428696549, rs1467819272, rs17855812, and so on) may play potential role in the procession of HCC carcinogenesis although they were excluded in the current study according to screening power. Fifth, although the effects of *ADAMTS4* SNPs on OS status were investigated among these patients with HCC, their effects on tumor occurrence‐free survival were not estimated. Additionally, more analyses for SNPs modifying *ADAMTS4* functions are not done, except for *ADAMTS4* expression based on tissular samples with HCC. Therefore, more functional and mechanical analyses deserve further elucidation on the basis of a larger sample size, more SNPs, and the combination of SNPs and liver carcinogenesis.

## 5 CONCLUSIONS

To the best of our knowledge, this is the first study to explore correlation between *ADAMTS4* SNPs and HCC risk and prognosis among Guangxi Population from a high incidence area of HCC. Some evidence was found to suggest that *ADAMTS4* rs538321148 and rs1014509103 polymorphisms may increase HCC risk and modify the prognosis of patients with HCC. Following confirmation in a large sample size (including meta‐analyses focused on this topic), these findings may be used to identify susceptible individuals at high risk of HCC, especially those with positive HBV infection and AFB1 exposure.

## CONFLICT OF INTEREST

None declared.

## Data Availability

The data that support the findings of this study are available on request from the corresponding author. The data are not publicly available due to privacy or ethical restrictions.
