# Supplementary material for: Single‐nucleotide polymorphisms in the coding region of a disintegrin and metalloproteinase with thrombospondin motifs 4 and hepatocellular carcinoma: A retrospective case‐control study
Source: Cancer Med. 2019 Oct 30;8(18):7869–80. doi: 10.1002/cam4.2646 (PMC6912020; doi:10.1002/cam4.2646)
Supplement: Supplementary file 1 [file CAM4-8-7869-s001.doc]

**Single-nucleotide polymorphisms in the coding region of a disintegrin and metalloproteinase with thrombospondin motifs 4 and hepatocellular carcinoma: A retrospective case-control study**

Xing-Zhizi Wang^1#^, Wei-Zhong Tang^2#^, Qun-Ying Su^1#^, Jin-Guang Yao^1^, Xiao-Ying Huang^1^, Qin-Qin Long^1^, Xue-Min Wu^1^, Qiang Xia^3^, Xi-Dai Long^1,3^

**Supplementary Material and Methods**

**Supplementary Table S1 – S4**

**Supplementary Figure S1.**

**Supplementary Materials and Methods**

**Study population**

The present study was approved by the ethic committees of the participating hospitals and was carried out in accordance with the approved guidelines (No. AYJM20090112). This is a hospital-based case-control study conducted in the affiliated hospitals of Youjiang Medical College for Nationalities and Guangxi Medical University in Guangxi Region, a major high-incidence area of HCC in China. Details regarding the Guangxiese HCC study, including the recruitment of study subjects, data collection, and descriptive information about the subjects in this study, have been previously described (1, 2). Briefly, all cases were patients diagnosed with histopathologically confirmed HCC in the aforementioned hospitals during January 2009 and December 2013. Both case and control recruitment are still ongoing. Control individuals without clinic evidence of hepatic diseases or tumors were recruited from the general-health check-up center of the same hospitals during the same period. To control the effects of confounders, cases were individually matched (1:1 or 1:2) to controls based on gender, age (±5 years), and ethnicity (Han, Zhuang). Every potential control was first surveyed by using a short question questionnaire to elicit willingness to participate in the study and provide preliminary demographic data for matching. In this study, a total of 862 cases and 1120 controls, representing 98% of eligible cases and 97% of eligible controls, were enrolled and interviewed. Among these subjects, we randomly selected 200-matched cases and controls to form the screening set and the remaining patients formed the validation set (Supplementary Fig. S1).

**Data and sample collection**

Informed consent was obtained from each participant before inclusion in the study. Subject evaluation included a structured interview that elicited information on demographic characteristics (including age, race, detailed medical history for themselves and their families, food-consumption history, and migration history); collection of study samples; and a standardized clinical examination. At the same time, 4 mL of peripheral blood was obtained for the analysis of serum albumin-aflatoxin B1 adducts (se-AAA) and single nucleotide polymorphisms (SNPs) of ADAMTS4. Surgically removed tumor samples of all cases were collected for ADAMTS4 protein expression assay. In addition, analyzing the levels of ADAMTS4 mRNA expression, we also collected 25 fresh cancer tissue specimens according to our published criteria (2). Clinical pathological data, including cirrhosis, tumor size, portal vein tumor (PVT), tumor stage, and medical treatment information, were obtained through the patients’ medical records. In this study, hepatitis B virus (HBV) and hepatitis C virus (HCV) infection status was ascertained by the hepatitis B surface antigen (HBsAg) and anti-HCV, respectively. Tumor stage and grade was elucidated according to WHO tumor, node, and metastasis system and Edmondson differentiation grading system, respectively; whereas liver cirrhosis was diagnosed by pathological examination.

For survival analysis, we followed all HCC cases and more detailed follow-up information was described in our previous studies (2). Briefly, all patients underwent serial monitoring every 2 months for the first 2 years and semiannually thereafter for detection of any recurrence. In this study, the last follow-up day was set on 31 July 2018, and survival status was ascertained by means of clinic records and patient or family contact. We defined the duration of the overall survival (OS) as from the date of curative treatment to the date of death or last known date alive.

**AFB1 information**

In this study, AFB1 exposure status was elucidated using se-AAA. The amount of se-AAA was tested by a comparative enzyme-linked immunosorbent assay (3). We defined se-AAA ≥ 2.18 ln fmol/mg as positive-AFB1 status according to our previous report (2).

**Single nucleotide polymorphisms (SNPs) selection and genotyping**

According to the prediction value of Sorting Intolerant from Tolerant (SIFT, http://sift.bii.a-star.edu.sg (4)) for SNPs in the coding region of ADAMTS4 and the criteria of SIFT less than 0.05, a total of 258 SNPs were selected for initial screening analyses using the SNaPshot method (Applied Biosystems [ABI], Foster City, CA) in the first stage study (Supplementary Table S1). In the second-stage study, six significant SNPs (rs150616368, rs538321148, rs17855812, rs1014509103, rs1485965919, and rs773407656) were selected after the first screening analyses according to the screening power and used to validate using a larger sample size (including 662 cases with HCC and 920 controls). Their genotypes were evaluated by the SNaPshot method (ABI).

For quality control, the controls and cases were genotyped at the same time, and duplicate test samples and two water samples (PCR negative controls) were included in each 96-well plate, with the technician blinded to the identity of the samples. Additionally, controls were included in each run, and repeated genotyping and sequencing of a random 5% subset yielded 100% identical genotypes.

**ADAMTS4 mRNA-expressing analysis**

Total RNA was extracted from cancerous tissue (n = 25) using E.Z.M.A.^TM^ MicroElute Total RNA Kit with DNase I (catalog#R6831-02, Omega Bio-tek, Inc., Norcross, GA, USA) and corresponding first-strand cDNA was synthesized using RevertAid^TM^ First Strand cDNA Synthesis Kit (catalog#K1622, Fermentas Inc., Glen Burnie, MD, USA). The relative quantitation of ADAMTS4 mRNA-expressing levels using the comparative CT method (2^-ΔΔCt^ method) was carried out by TaqMan-PCR (with an internal control UBC mRNA). The PCR primers and TaqMans were 5’-CTTCCT GGACA ATGGC TATGG-3’, 5’-GGAAA GTCAC AGGCA GATGC A-3’, and 5’-FAM-TTAGA CAAAC CAGAG GCT-MGB-3’ for ADAMTS4 and 5’-GGGCA CTGGT TTTCTT TCCA-3’, 5’-CGCCG AGAAG GGACT ACTTT T-3’, and 5’-HEX-AGAGCGGAACAGGC-MGB-3’ (2). PCR amplification was performed in a 25-µL final reaction mixture containing 1 × Premix Ex Taq^TM^ (TaKaRa Biotechnology (Dalian) Co., Ltd., Dalian, China), 1 × TaqMan® Gene Expression Assay Mix (cat#4331182, ABI), and 1µL reverse transcription product with cDNAs. PCR reaction conditions comprised an initial step at 95 ℃ for 2 min, followed by 45 cycles at 95 ℃ for 10 sec and 60 ℃ for 1 min. Data analysis for the relative level of ADAMTS4 mRNA expression was performed with the iQ^TM^ Optical System software Version 2.0 (Bio-Rad).

**ADAMTS4 protein-expressing assay**

The expression levels of ADAMTS4 protein were analyzed by immunohistochemistry in tissue slides, as previously described (5). The corresponding anti-ADAMTS4 polyclonal antibody (dilution 1:250, catalog# O75173) and HRP-conjugated secondary antibody (catalog# KIT-9705) were obtained from AVIVA Systems Biology, Inc. (San Diego, California) and Maixin Biotechnology, Inc. (Xiamen, China), respectively. In this study, ADAMTS4 protein-expressing levels were divided into three classifications: low (the immunoreactive score (IRS), < 4), medium (IRS, 4 - 8), and high (IRS, > 8), according to the value of IRS systems (6).

**Statistical analysis**

The distribute difference between groups was tested by chi-square test, *t* test, or analysis of variance (ANOVA) test. In this study, the test for screening and validating the main effects of ADAMTS4 SNPs was based on the additive model, treating genotype as an ordinal variable (wild type coded as 0, heterozygote as 1, and homozygotes variant as 2). The correction for multiple testing in the screen stage was done using the correlation matrix-based method (7), which considers the linkage disequilibrium between SNPs. The effective number of independent SNPs was determined using the spectral decomposition, and *P*-value < 1.94 × 10^-4^ was regarded as significant threshold for the main effects of SNPs. Based on individually matched design, we did conditional logistic regression (including all known causes of Guangxiese HCC) to estimate odds ratios (ORs) for risk of HCC and their 95% confidence intervals (CIs).

Spearman’s *r* correlation analyses were used to test the association between ADATMS4 SNPs and the amount of ADAMTS4 protein. The effects of ADAMTS4 SNPs on the OS of HCC were elucidated by Kaplan–Meier survival model (with *Log-Rank* test) and the Cox’s multivariate regression model (with step-wise forward selection based on the likelihood ratio test). Then, hazard ratio (HR) and corresponding 95% CI was calculated in the same Cox’s regression model (including all significant variables). All statistical analyses were performed using the SPSS version 18 (SPSS Institute, Chicago, IL, USA).

**Supplementary Reference**

1. Long XD, Zhao D, Wang C, Huang XY, Yao JG, Ma Y, et al. Genetic Polymorphisms in DNA Repair Genes XRCC4 and XRCC5 and Aflatoxin B1-related Hepatocellular Carcinoma. Epidemiology. 2013;24(5):671-81.

2. Long XD, Yao JG, Zeng Z, Ma Y, Huang XY, Wei ZH, et al. Polymorphisms in the coding region of X-ray repair complementing group 4 and aflatoxin B1-related hepatocellular carcinoma. Hepatology. 2013;58(1):171-81.

3. Scholl PF, Groopman JD. Long-term stability of human aflatoxin B1 albumin adducts assessed by isotope dilution mass spectrometry and high-performance liquid chromatography-fluorescence. Cancer epidemiology, biomarkers & prevention : a publication of the American Association for Cancer Research, cosponsored by the American Society of Preventive Oncology. 2008;17(6):1436-9.

4. Ng PC, Henikoff S. SIFT: Predicting amino acid changes that affect protein function. Nucleic Acids Res. 2003;31(13):3812-4.

5. Long XD, Ma Y, Zhou YF, Ma AM, Fu GH. Polymorphism in xeroderma pigmentosum complementation group C codon 939 and aflatoxin B1-related hepatocellular carcinoma in the Guangxi population. Hepatology. 2010;52(4):1301-9.

6. Friedrichs K, Gluba S, Eidtmann H, Jonat W. Overexpression of p53 and prognosis in breast cancer. Cancer. 1993;72(12):3641-7.

7. Nyholt DR. A simple correction for multiple testing for single-nucleotide polymorphisms in linkage disequilibrium with each other. American journal of human genetics. 2004;74(4):765-9.

**Supplementary Figures**


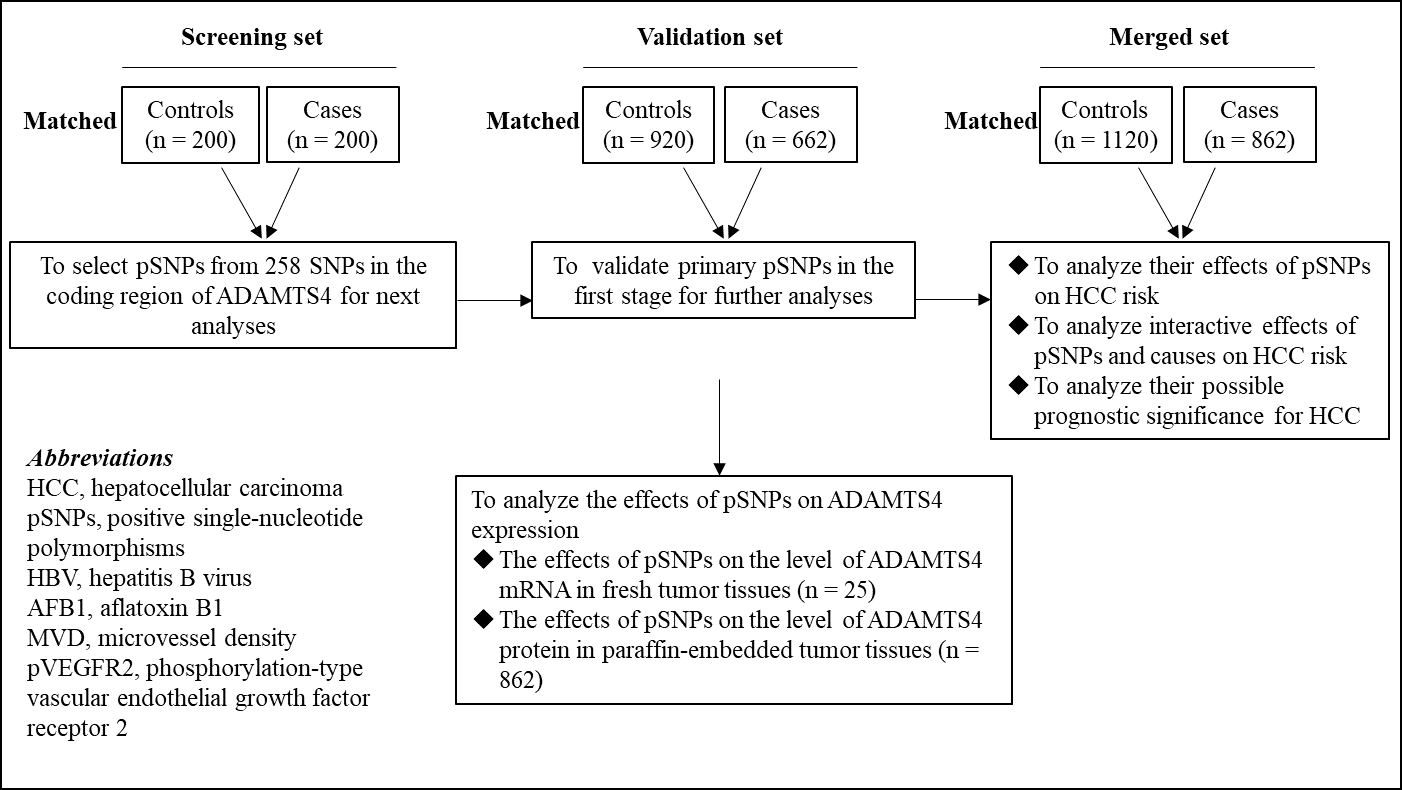


**Supplementary Figure S1**. The study designs. This study is a hospital-based cases-control study and consists of 862 cases with pathologically diagnosed hepatocellular carcinoma (HCC) and 1120 controls without any evidence of liver diseases. Positive single-nucleotide polymorphism (SNPs) in the coding region of ADAMTS4 were first screened and validated via two stage analyses, and then two SNPs were ultimately selected for analyzing their effects on HCC risk and prognosis.

**Supplementary Table S**1 Demographic and Etiologic Characteristics of HCC Cases and Controls

|  |  | Screening Set | |  | Validation Set | |  | Merged Set | | |
| --- | --- | --- | --- | --- | --- | --- | --- | --- | --- | --- |
| Variable |  | Controls | HCCs |  | Controls | HCCs |  | Controls | HCCs | OR(95%CI/*P*_trend_) |
| Total |  | 200 | 200 |  | 920 | 662 |  | 1120 | 862 |  |
| Sex |  |  |  |  |  |  |  |  |  |  |
| Male |  | 143 | 143 |  | 703 | 486 |  | 846 | 629 | Reference |
| Female |  | 57 | 57 |  | 217 | 176 |  | 274 | 233 | 0.88 (0.69-1.12/0.29) |
| Age (years) |  |  |  |  |  |  |  |  |  |  |
| ≤34 |  | 25 | 25 |  | 97 | 58 |  | 122 | 83 | Reference |
| 35-40 |  | 46 | 46 |  | 123 | 104 |  | 169 | 150 | 1.41 (0.93-2.14/0.11) |
| 41-45 |  | 36 | 36 |  | 126 | 87 |  | 162 | 123 | 1.15 (0.75-1.77/0.51) |
| 46-50 |  | 31 | 31 |  | 128 | 100 |  | 159 | 131 | 1.32 (0.86-2.03/0.20) |
| 51-55 |  | 20 | 20 |  | 121 | 81 |  | 141 | 101 | 0.99 (0.64-1.54/0.97) |
| 56-60 |  | 13 | 13 |  | 112 | 83 |  | 125 | 96 | 1.38 (0.88-2.16/0.16) |
| 61-65 |  | 7 | 7 |  | 119 | 84 |  | 126 | 91 | 1.10 (0.70-1.74/0.68) |
| ≥66 |  | 22 | 22 |  | 94 | 65 |  | 116 | 87 | 1.21 (0.76-1.92/0.42) |
| Ethnicity |  |  |  |  |  |  |  |  |  |  |
| Han |  | 98 | 98 |  | 458 | 316 |  | 556 | 414 | Reference |
| Zhuang |  | 102 | 102 |  | 462 | 346 |  | 564 | 448 | 0.99 (0.80-1.23/0.95) |
| Smoking status |  |  |  |  |  |  |  |  |  |  |
| No |  | 122 | 124 |  | 706 | 521 |  | 828 | 645 | Reference |
| Yes |  | 78 | 76 |  | 214 | 141 |  | 292 | 217 | 0.85 (0.29-2.46/0.76) |
| Drinking status |  |  |  |  |  |  |  |  |  |  |
| No |  | 116 | 121 |  | 709 | 523 |  | 825 | 644 | Reference |
| Yes |  | 84 | 79 |  | 211 | 139 |  | 295 | 218 | 0.93 (0.32-2.71/0.90) |
| HBV infection |  |  |  |  |  |  |  |  |  |  |
| Negative |  | 129 | 45 |  | 687 | 174 |  | 816 | 219 | Reference |
| Positive |  | 71 | 155 |  | 233 | 488 |  | 304 | 643 | 7.88 (6.36-9.77/2.36×10^-79^) |
| HCV infection |  |  |  |  |  |  |  |  |  |  |
| Negative |  | 173 | 160 |  | 747 | 543 |  | 920 | 703 | Reference |
| Positive |  | 27 | 40 |  | 173 | 119 |  | 200 | 159 | 1.23 (0.93-1.62/0.15) |
| AFB1 exposure |  |  |  |  |  |  |  |  |  |  |
| Negative |  | 103 | 40 |  | 483 | 156 |  | 586 | 196 | Reference |
| Positive |  | 97 | 160 |  | 437 | 506 |  | 534 | 666 | 3.52 (2.81-4.40/2.07×10^-28^) |

**Supplementary Table S**2 Joint effects of HBV and AFB1 status on HCC risk

|  |  | Controls | |  | HCCs | |  |  |  |
| --- | --- | --- | --- | --- | --- | --- | --- | --- | --- |
| Variables |  | n | % |  | n | % |  | OR (95% CI) ^a^ | *P*_trend_ |
| HBV (-)/AFB1 (-) |  | 423 | 37.8 |  | 65 | 7.5 |  | Reference |  |
| AFB1 (+) |  | 393 | 35.1 |  | 154 | 17.9 |  | 2.54 (1.85-3.51) | 1.09×10^-8^ |
| HBV (+)/AFB1 (-) |  | 163 | 14.6 |  | 131 | 15.2 |  | 5.23 (3.70-7.42) | 1.22×10^-20^ |
| AFB1 (+) |  | 141 | 12.6 |  | 512 | 59.4 |  | 23.64 (17.15-32.58) | 3.72×10^-83^ |

^a^ OR conditional on matched set.

**Supplementary Table S3 The polymorphisms information of ADAMTS4 and their distribution among cases with hepatocellular carcinoma (HCC) and controls**

| NO. | dbSNP rs# | Chr:bp | Allele  (X/Y)^a^ | Genotype  (XX/XY/YY)^b^ | AA  position | AA  Change | SIFT |  | Controls  (N_XX_/N_XY_/N_YY_)^c^ | HCCs  (N_XX_/N_XY_/N_YY_)^c^ | *P* |
| --- | --- | --- | --- | --- | --- | --- | --- | --- | --- | --- | --- |
| SNP001 | rs1387725777 | 1:161198609 | G/A | GG/GA/AA | 7 | H/Y | 0.02 |  | 184/13/3 | 185/11/4 | 8.52E-01 |
| SNP002 | rs1370245538 | 1:161198605 | G/A | GG/GA/AA | 8 | P/L | 0 |  | 181/15/4 | 182/14/4 | 8.63E-01 |
| SNP003 | rs779009777 | 1:161198574 | C/G | CC/CG/GG | 18 | W/C | 0.01 |  | 171/29/0 | 173/25/2 | 7.73E-01 |
| SNP004 | rs994679375 | 1:161198567 | G/C | GG/GC/CC | 21 | Q/E | 0.05 |  | 164/32/4 | 132/41/27 | 2.65E-04 |
| SNP005 | rs1211815395 | 1:161198558 | G/A | GG/GA/AA | 24 | L/F | 0.05 |  | 161/34/5 | 143/46/11 | 3.51E-02 |
| SNP006 | rs1488208820 | 1:161198554 | A/C | AA/AC/CC | 25 | L/R | 0.01 |  | 180/20/0 | 178/21/1 | 7.44E-01 |
| SNP007 | rs1192093944 | 1:161198549 | G/T | GG/GT/TT | 27 | P/T | 0.01 |  | 185/15/0 | 186/14/0 | 8.47E-01 |
| SNP008 | rs150616368 | 1:161198539 | G/A | GG/GA/AA | 30 | P/L | 0 |  | 153/41/6 | 108/52/40 | 2.30E-06 |
| SNP009 | rs895122569 | 1:161198503 | A/G | AA/AG/GG | 42 | L/P | 0.03 |  | 186/14/0 | 184/16/0 | 7.04E-01 |
| SNP010 | rs1482491358 | 1:161198456 | C/G | CC/CG/GG | 58 | E/Q | 0.05 |  | 172/25/3 | 155/33/12 | 2.78E-02 |
| SNP011 | rs1201925530 | 1:161198453 | C/T | CC/CT/TT | 59 | E/K | 0.03 |  | 182/18/0 | 177/23/0 | 4.10E-01 |
| SNP012 | rs771427107 | 1:161198450 | C/G | CC/CG/GG | 60 | E/Q | 0 |  | 190/10/0 | 191/9/0 | 8.14E-01 |
| SNP013 | rs1343794855 | 1:161198444 | C/T | CC/CT/TT | 62 | V/M | 0 |  | 191/9/0 | 190/10/0 | 8.14E-01 |
| SNP014 | rs916853796 | 1:161198428 | A/G | AA/AG/GG | 67 | L/P | 0 |  | 189/11/0 | 184/16/0 | 3.19E-01 |
| SNP015 | rs1350028451 | 1:161198429 | G/A | GG/GA/AA | 67 | L/F | 0.03 |  | 173/22/5 | 155/36/9 | 1.91E-02 |
| SNP016 | rs1479070493 | 1:161198426 | T/G | TT/TG/GG | 68 | N/H | 0.01 |  | 183/14/3 | 167/31/2 | 1.56E-02 |
| SNP017 | rs1451103538 | 1:161198395 | G/A | GG/GA/AA | 78 | P/L | 0.05 |  | 172/26/2 | 154/42/4 | 2.05E-02 |
| SNP018 | rs373458115 | 1:161198378 | G/A | GG/GA/AA | 84 | R/C | 0 |  | 188/12/0 | 187/13/0 | 8.36E-01 |
| SNP019 | rs1181446997 | 1:161198369 | C/T | CC/CT/TT | 87 | A/T | 0.02 |  | 186/14/0 | 171/28/1 | 1.55E-02 |
| SNP020 | rs1162569247 | 1:161198333 | C/T | CC/CT/TT | 99 | D/N | 0 |  | 181/19/0 | 185/15/0 | 4.73E-01 |
| SNP021 | rs538321148 | 1:161198318 | C/T | CC/CT/TT | 104 | V/I | 0 |  | 192/8/0 | 183/17/0 | 6.30E-02 |
| SNP022 | rs903882429 | 1:161198251 | G/A | GG/GA/AA | 126 | T/I | 0 |  | 130/62/8 | 84/90/26 | 4.00E-06 |
| SNP023 | rs1000852820 | 1:161198252 | T/C | TT/TC/CC | 126 | T/A | 0.05 |  | 172/25/3 | 156/32/12 | 3.73E-02 |
| SNP024 | rs747170337 | 1:161198243 | T/C | TT/TC/CC | 129 | T/A | 0.04 |  | 166/30/4 | 164/32/4 | 7.92E-01 |
| SNP025 | rs780270695 | 1:161198239 | C/A | CC/CA/AA | 130 | G/V | 0 |  | 193/7/0 | 190/10/0 | 4.57E-01 |
| SNP026 | rs745957886 | 1:161198236 | G/A | GG/GA/AA | 131 | T/I | 0.04 |  | 173/26/1 | 154/43/3 | 1.39E-02 |
| SNP027 | rs778900326 | 1:161198230 | T/C | TT/TC/CC | 133 | N/S | 0.01 |  | 184/16/0 | 183/17/0 | 8.56E-01 |
| SNP028 | rs199826453 | 1:161198221 | G/A | GG/GA/AA | 136 | P/L | 0 |  | 192/8/0 | 191/9/0 | 8.04E-01 |
| SNP029 | rs749780664 | 1:161198215 | G/A | GG/GA/AA | 138 | S/L | 0 |  | 194/6/0 | 192/8/0 | 5.86E-01 |
| SNP030 | rs1204212139 | 1:161198216 | A/G | AA/AG/GG | 138 | S/P | 0 |  | 193/7/0 | 196/4/0 | 3.59E-01 |
| SNP031 | rs1165644171 | 1:161198203 | A/G | AA/AG/GG | 142 | L/P | 0 |  | 195/5/0 | 196/4/0 | 7.36E-01 |
| SNP032 | rs1280794625 | 1:161198198 | A/C | AA/AC/CC | 144 | W/G | 0.01 |  | 186/14/0 | 182/18/0 | 4.61E-01 |
| SNP033 | rs1176787186 | 1:161198185 | G/T | GG/GT/TT | 148 | A/D | 0 |  | 193/7/0 | 194/6/0 | 7.78E-01 |
| SNP034 | rs562873566 | 1:161198182 | A/G | AA/AG/GG | 149 | L/P | 0 |  | 154/38/8 | 157/34/9 | 7.18E-01 |
| SNP035 | rs1428696549 | 1:161198176 | C/T | CC/CT/TT | 151 | G/D | 0 |  | 199/1/0 | 189/11/0 | 3.38E-03 |
| SNP036 | rs764698455 | 1:161198174 | C/T | CC/CT/TT | 152 | V/M | 0 |  | 181/18/1 | 174/19/7 | 2.68E-01 |
| SNP037 | rs984084303 | 1:161198137 | G/A | GG/GA/AA | 164 | P/L | 0 |  | 192/8/0 | 188/12/0 | 3.59E-01 |
| SNP038 | rs1252001488 | 1:161198138 | G/A | GG/GA/AA | 164 | P/S | 0 |  | 161/36/3 | 167/24/9 | 4.35E-01 |
| SNP039 | rs1412762783 | 1:161198093 | G/A | GG/GA/AA | 179 | H/Y | 0 |  | 190/10/0 | 187/13/0 | 5.19E-01 |
| SNP040 | rs764740290 | 1:161198084 | G/A | GG/GA/AA | 182 | R/C | 0 |  | 181/19/0 | 176/21/3 | 4.20E-01 |
| SNP041 | rs761754528 | 1:161198080 | C/T | CC/CT/TT | 183 | R/Q | 0.02 |  | 173/26/1 | 164/34/2 | 2.17E-01 |
| SNP042 | rs376506791 | 1:161198081 | G/A | GG/GA/AA | 183 | R/W | 0 |  | 179/21/0 | 181/18/1 | 7.39E-01 |
| SNP043 | rs1465008508 | 1:161198076 | C/A | CC/CA/AA | 184 | K/N | 0.03 |  | 153/39/8 | 156/34/10 | 7.20E-01 |
| SNP044 | rs770389695 | 1:161198056 | C/A | CC/CA/AA | 191 | G/V | 0.01 |  | 166/34/0 | 142/48/10 | 4.35E-03 |
| SNP045 | rs377253620 | 1:161198042 | C/T | CC/CT/TT | 196 | V/I | 0.04 |  | 181/17/2 | 157/42/1 | 9.14E-04 |
| SNP046 | rs1000963834 | 1:161198002 | C/T | CC/CT/TT | 209 | R/K | 0.02 |  | 163/35/2 | 140/49/11 | 7.29E-03 |
| SNP047 | rs752664084 | 1:161197999 | G/A | GG/GA/AA | 210 | A/V | 0.02 |  | 161/35/4 | 150/38/12 | 1.86E-01 |
| SNP048 | rs1343826431 | 1:161196880 | G/A | GG/GA/AA | 212 | R/C | 0 |  | 183/16/1 | 178/22/0 | 3.99E-01 |
| SNP049 | rs1396183287 | 1:161196857 | A/C | AA/AC/CC | 219 | F/L | 0.01 |  | 182/18/0 | 164/32/4 | 8.45E-03 |
| SNP050 | rs1392380959 | 1:161196859 | A/G | AA/AG/GG | 219 | F/L | 0.01 |  | 185/14/1 | 181/17/2 | 4.73E-01 |
| SNP051 | rs116305764 | 1:161196847 | G/C | GG/GC/CC | 223 | L/V | 0 |  | 162/37/1 | 157/43/0 | 5.34E-01 |
| SNP052 | rs142929819 | 1:161196843 | A/G | AA/AG/GG | 224 | V/A | 0 |  | 173/27/0 | 165/34/1 | 2.69E-01 |
| SNP053 | rs1468021009 | 1:161196840 | A/T | AA/AT/TT | 225 | V/E | 0 |  | 183/17/0 | 178/22/0 | 3.99E-01 |
| SNP054 | rs766240688 | 1:161196811 | C/T | CC/CT/TT | 235 | G/S | 0.01 |  | 173/23/4 | 147/48/5 | 1.15E-03 |
| SNP055 | rs1327205041 | 1:161196786 | A/G | AA/AG/GG | 243 | L/P | 0 |  | 152/39/9 | 157/34/9 | 5.51E-01 |
| SNP056 | rs1398314770 | 1:161196783 | G/A | GG/GA/AA | 244 | T/I | 0 |  | 173/23/4 | 174/23/3 | 8.83E-01 |
| SNP057 | rs1423800000 | 1:161196776 | C/T | CC/CT/TT | 246 | M/I | 0.03 |  | 178/21/1 | 183/16/1 | 3.99E-01 |
| SNP058 | rs1211420229 | 1:161196769 | C/T | CC/CT/TT | 249 | A/T | 0.03 |  | 158/37/5 | 167/29/4 | 2.49E-01 |
| SNP059 | rs1467819272 | 1:161196763 | T/C | TT/TC/CC | 251 | K/E | 0 |  | 183/17/0 | 159/31/10 | 6.54E-04 |
| SNP060 | rs909895361 | 1:161196759 | G/T | GG/GT/TT | 252 | A/D | 0 |  | 174/24/2 | 166/34/0 | 2.63E-01 |
| SNP061 | rs1421370467 | 1:161196754 | T/C | TT/TC/CC | 254 | K/E | 0 |  | 163/34/3 | 180/16/4 | 3.43E-03 |
| SNP062 | rs1361853607 | 1:161196751 | G/A | GG/GA/AA | 255 | H/Y | 0.01 |  | 159/40/1 | 175/23/2 | 3.11E-02 |
| SNP063 | rs1167201437 | 1:161196735 | T/C | TT/TC/CC | 260 | N/S | 0 |  | 180/18/2 | 161/29/10 | 7.38E-03 |
| SNP064 | rs984016369 | 1:161196722 | C/A | CC/CA/AA | 264 | L/F | 0 |  | 189/11/0 | 178/22/0 | 4.56E-02 |
| SNP065 | rs779672378 | 1:161196714 | G/C | GG/GC/CC | 267 | T/S | 0.01 |  | 190/10/0 | 187/13/0 | 5.19E-01 |
| SNP066 | rs1301940748 | 1:161196715 | T/A | TT/TA/AA | 267 | T/S | 0.01 |  | 163/36/1 | 180/16/4 | 1.50E-02 |
| SNP067 | rs758269430 | 1:161196711 | C/T | CC/CT/TT | 268 | R/Q | 0.01 |  | 186/14/0 | 191/9/0 | 2.83E-01 |
| SNP068 | rs1350145239 | 1:161196712 | G/A | GG/GA/AA | 268 | R/W | 0 |  | 150/41/9 | 156/33/11 | 4.79E-01 |
| SNP069 | rs750305241 | 1:161196708 | A/T | AA/AT/TT | 269 | L/Q | 0 |  | 162/37/1 | 182/16/2 | 3.95E-03 |
| SNP070 | rs1230848540 | 1:161196682 | C/G | CC/CG/GG | 278 | G/R | 0 |  | 184/15/1 | 195/5/0 | 1.37E-02 |
| SNP071 | rs753840573 | 1:161196673 | C/T | CC/CT/TT | 281 | V/M | 0 |  | 187/13/0 | 177/23/0 | 8.06E-02 |
| SNP072 | rs764256767 | 1:161196669 | C/T | CC/CT/TT | 282 | G/E | 0.01 |  | 184/13/3 | 172/25/3 | 5.52E-02 |
| SNP073 | rs992660039 | 1:161196657 | G/A | GG/GA/AA | 286 | A/V | 0 |  | 162/36/2 | 184/16/0 | 1.29E-03 |
| SNP074 | rs1249974188 | 1:161196651 | G/C | GG/GC/CC | 288 | T/S | 0.02 |  | 164/30/6 | 184/14/2 | 2.94E-03 |
| SNP075 | rs201261893 | 1:161196645 | C/T | CC/CT/TT | 290 | R/H | 0.02 |  | 158/38/4 | 169/27/4 | 1.54E-01 |
| SNP076 | rs1243925734 | 1:161196646 | G/A | GG/GA/AA | 290 | R/C | 0 |  | 186/14/0 | 170/29/1 | 1.06E-02 |
| SNP077 | rs759878984 | 1:161196643 | T/C | TT/TC/CC | 291 | S/G | 0 |  | 179/20/1 | 170/26/4 | 1.77E-01 |
| SNP078 | rs148385833 | 1:161196625 | G/A | GG/GA/AA | 297 | R/W | 0 |  | 150/42/8 | 154/36/10 | 6.40E-01 |
| SNP079 | rs1270292641 | 1:161196618 | A/G | AA/AG/GG | 299 | L/P | 0.02 |  | 163/35/2 | 181/15/4 | 9.49E-03 |
| SNP080 | rs1324139807 | 1:161196615 | T/C | TT/TC/CC | 300 | N/S | 0 |  | 191/9/0 | 183/17/0 | 1.05E-01 |
| SNP081 | rs17855813 | 1:161196604 | C/T | CC/CT/TT | 304 | D/N | 0 |  | 178/20/2 | 164/28/8 | 4.68E-02 |
| SNP082 | rs746852468 | 1:161196600 | G/A | GG/GA/AA | 305 | S/L | 0.01 |  | 193/7/0 | 182/17/1 | 2.31E-02 |
| SNP083 | rs754121205 | 1:161196589 | G/T | GG/GT/TT | 309 | H/N | 0 |  | 179/19/2 | 163/27/10 | 2.31E-02 |
| SNP084 | rs972154060 | 1:161196576 | G/C | GG/GC/CC | 313 | A/G | 0 |  | 165/34/1 | 176/24/0 | 1.21E-01 |
| SNP085 | rs1412877986 | 1:161196567 | A/G | AA/AG/GG | 316 | F/S | 0 |  | 166/30/4 | 180/18/2 | 4.05E-02 |
| SNP086 | rs1355620120 | 1:161196564 | G/A | GG/GA/AA | 317 | T/I | 0 |  | 192/8/0 | 186/14/0 | 1.88E-01 |
| SNP087 | rs768005913 | 1:161196562 | G/A | GG/GA/AA | 318 | R/C | 0 |  | 166/34/0 | 185/15/0 | 3.76E-03 |
| SNP088 | rs927128980 | 1:161196301 | G/T | GG/GT/TT | 320 | D/E | 0 |  | 198/2/0 | 197/3/0 | 6.53E-01 |
| SNP089 | rs1469077329 | 1:161196294 | C/T | CC/CT/TT | 323 | G/R | 0 |  | 183/16/1 | 164/34/2 | 5.08E-03 |
| SNP090 | rs748391228 | 1:161196279 | C/T | CC/CT/TT | 328 | D/N | 0.02 |  | 151/43/6 | 153/38/9 | 8.15E-01 |
| SNP091 | rs781204896 | 1:161196275 | G/A | GG/GA/AA | 329 | T/M | 0 |  | 179/21/0 | 161/38/1 | 1.17E-02 |
| SNP092 | rs780594948 | 1:161196269 | C/T | CC/CT/TT | 331 | G/D | 0 |  | 194/6/0 | 179/20/1 | 2.80E-03 |
| SNP093 | rs758870997 | 1:161196264 | C/A | CC/CA/AA | 333 | A/S | 0 |  | 196/4/0 | 197/3/0 | 4.11E-01 |
| SNP094 | rs765546125 | 1:161196260 | T/C | TT/TC/CC | 334 | D/G | 0.01 |  | 165/33/2 | 186/13/1 | 1.36E-03 |
| SNP095 | rs752403013 | 1:161196251 | G/A | GG/GA/AA | 337 | T/I | 0 |  | 153/43/4 | 152/37/11 | 9.06E-01 |
| SNP096 | rs137881629 | 1:161196239 | G/A | GG/GA/AA | 341 | P/L | 0.03 |  | 184/14/2 | 173/23/4 | 7.58E-02 |
| SNP097 | rs766307909 | 1:161196240 | G/C | GG/GC/CC | 341 | P/A | 0 |  | 162/34/4 | 184/15/1 | 1.29E-03 |
| SNP098 | rs773010345 | 1:161196234 | G/A | GG/GA/AA | 343 | R/W | 0 |  | 193/6/1 | 190/10/0 | 4.57E-01 |
| SNP099 | rs1399365047 | 1:161196230 | C/T | CC/CT/TT | 344 | S/N | 0 |  | 189/10/1 | 181/18/1 | 1.29E-01 |
| SNP100 | rs1462710363 | 1:161196221 | A/G | AA/AG/GG | 347 | I/T | 0 |  | 165/35/0 | 166/34/0 | 8.95E-01 |
| SNP101 | rs1240732446 | 1:161196199 | C/G | CC/CG/GG | 354 | Q/H | 0.01 |  | 179/20/1 | 158/38/4 | 3.95E-03 |
| SNP102 | rs539285871 | 1:161196195 | C/A | CC/CA/AA | 356 | A/S | 0.01 |  | 179/21/0 | 194/6/0 | 2.80E-03 |
| SNP103 | rs1259824876 | 1:161196174 | G/C | GG/GC/CC | 363 | L/V | 0 |  | 185/15/0 | 179/20/1 | 2.95E-01 |
| SNP104 | rs1404765211 | 1:161195632 | T/A | TT/TA/AA | 365 | H/L | 0 |  | 189/11/0 | 180/18/2 | 9.24E-02 |
| SNP105 | rs17855812 | 1:161195621 | T/C | TT/TC/CC | 369 | M/V | 0 |  | 174/24/2 | 138/57/5 | 1.39E-05 |
| SNP106 | rs1372002191 | 1:161195615 | G/T | GG/GT/TT | 371 | H/N | 0 |  | 183/16/1 | 166/34/0 | 1.08E-02 |
| SNP107 | rs779544957 | 1:161195555 | T/C | TT/TC/CC | 391 | M/V | 0 |  | 167/25/8 | 187/13/0 | 1.72E-03 |
| SNP108 | rs115845862 | 1:161195551 | G/A | GG/GA/AA | 392 | A/V | 0 |  | 172/26/2 | 155/39/6 | 2.78E-02 |
| SNP109 | rs140088846 | 1:161195528 | G/C | GG/GC/CC | 400 | P/A | 0.03 |  | 166/33/1 | 152/48/0 | 8.29E-02 |
| SNP110 | rs371451268 | 1:161195509 | G/A | GG/GA/AA | 406 | P/L | 0.02 |  | 169/28/3 | 184/15/1 | 1.99E-02 |
| SNP111 | rs994281707 | 1:161195500 | G/A | GG/GA/AA | 409 | A/V | 0 |  | 195/5/0 | 186/14/0 | 3.44E-02 |
| SNP112 | rs772396585 | 1:161195501 | C/A | CC/CA/AA | 409 | A/S | 0.03 |  | 187/13/0 | 179/21/0 | 1.51E-01 |
| SNP113 | rs539344608 | 1:161195488 | G/A | GG/GA/AA | 413 | T/I | 0 |  | 180/18/2 | 169/30/1 | 9.91E-02 |
| SNP114 | rs1236324495 | 1:161195489 | T/A | TT/TA/AA | 413 | T/S | 0 |  | 154/41/5 | 156/43/1 | 8.11E-01 |
| SNP115 | rs1462758214 | 1:161195479 | A/G | AA/AG/GG | 416 | L/P | 0 |  | 182/16/2 | 165/34/1 | 1.22E-02 |
| SNP116 | rs1203274438 | 1:161195476 | T/C | TT/TC/CC | 417 | D/G | 0 |  | 198/2/0 | 183/17/0 | 4.22E-04 |
| SNP117 | rs1263766946 | 1:161195477 | C/T | CC/CT/TT | 417 | D/N | 0 |  | 184/16/0 | 190/10/0 | 2.24E-01 |
| SNP118 | rs749833445 | 1:161195474 | T/G | TT/TG/GG | 418 | N/H | 0.02 |  | 167/33/0 | 154/46/0 | 1.03E-01 |
| SNP119 | rs770283440 | 1:161195471 | C/T | CC/CT/TT | 419 | G/S | 0 |  | 171/25/4 | 154/40/6 | 2.94E-02 |
| SNP120 | rs748974903 | 1:161194218 | T/C | TT/TC/CC | 422 | H/R | 0 |  | 198/2/0 | 195/5/0 | 2.53E-01 |
| SNP121 | rs1431059247 | 1:161194212 | A/C | AA/AC/CC | 424 | L/R | 0 |  | 188/12/0 | 186/14/0 | 6.85E-01 |
| SNP122 | rs763483506 | 1:161194205 | G/C | GG/GC/CC | 426 | D/E | 0 |  | 155/45/0 | 152/47/1 | 7.23E-01 |
| SNP123 | rs1445240861 | 1:161194179 | G/A | GG/GA/AA | 435 | P/L | 0.02 |  | 170/25/5 | 153/40/7 | 3.11E-02 |
| SNP124 | rs1298269593 | 1:161194180 | G/A | GG/GA/AA | 435 | P/S | 0.02 |  | 174/25/1 | 183/17/0 | 1.46E-01 |
| SNP125 | rs745567847 | 1:161194168 | G/A | GG/GA/AA | 439 | P/S | 0.01 |  | 185/14/1 | 192/8/0 | 1.33E-01 |
| SNP126 | rs868103024 | 1:161194164 | C/T | CC/CT/TT | 440 | G/D | 0 |  | 167/30/3 | 186/13/1 | 3.18E-03 |
| SNP127 | rs76156735 | 1:161194146 | T/G | TT/TG/GG | 446 | D/A | 0 |  | 173/23/4 | 159/37/4 | 6.24E-02 |
| SNP128 | rs778329496 | 1:161194144 | G/A | GG/GA/AA | 447 | R/C | 0 |  | 184/16/0 | 174/26/0 | 1.03E-01 |
| SNP129 | rs569554491 | 1:161194137 | C/T | CC/CT/TT | 449 | C/Y | 0 |  | 177/22/1 | 163/36/1 | 4.99E-02 |
| SNP130 | rs1261521074 | 1:161194122 | C/T | CC/CT/TT | 454 | G/E | 0 |  | 156/40/4 | 158/42/0 | 8.08E-01 |
| SNP131 | rs767364588 | 1:161194123 | C/T | CC/CT/TT | 454 | G/R | 0 |  | 186/14/0 | 189/11/0 | 5.35E-01 |
| SNP132 | rs143490869 | 1:161194111 | G/A | GG/GA/AA | 458 | R/C | 0 |  | 176/24/0 | 185/15/0 | 1.29E-01 |
| SNP133 | rs375012153 | 1:161194101 | G/A | GG/GA/AA | 461 | P/L | 0.01 |  | 167/31/2 | 185/15/0 | 5.61E-03 |
| SNP134 | rs1405372539 | 1:161194102 | G/A | GG/GA/AA | 461 | P/S | 0.01 |  | 174/25/1 | 171/29/0 | 6.63E-01 |
| SNP135 | rs1421823180 | 1:161194083 | C/A | CC/CA/AA | 467 | C/F | 0.03 |  | 189/11/0 | 188/12/0 | 8.30E-01 |
| SNP136 | rs1406403301 | 1:161194084 | A/G | AA/AG/GG | 467 | C/R | 0 |  | 157/39/4 | 158/41/1 | 9.03E-01 |
| SNP137 | rs1220008538 | 1:161194065 | G/A | GG/GA/AA | 473 | S/F | 0.05 |  | 173/26/1 | 164/34/2 | 2.17E-01 |
| SNP138 | rs1229877917 | 1:161194057 | G/A | GG/GA/AA | 476 | L/F | 0.03 |  | 174/26/0 | 163/35/2 | 1.31E-01 |
| SNP139 | rs545646079 | 1:161194032 | G/T | GG/GT/TT | 484 | T/N | 0.05 |  | 158/39/3 | 176/23/1 | 1.53E-02 |
| SNP140 | rs765392088 | 1:161194012 | C/T | CC/CT/TT | 491 | D/N | 0 |  | 190/10/0 | 188/12/0 | 6.61E-01 |
| SNP141 | rs1014509103 | 1:161194005 | G/A | GG/GA/AA | 493 | T/I | 0 |  | 155/37/8 | 112/54/34 | 5.03E-06 |
| SNP142 | rs199775251 | 1:161193996 | C/G | CC/CG/GG | 496 | G/A | 0.04 |  | 157/42/1 | 136/53/11 | 1.77E-02 |
| SNP143 | rs777208732 | 1:161193997 | C/T | CC/CT/TT | 496 | G/R | 0.01 |  | 178/22/0 | 162/37/1 | 2.51E-02 |
| SNP144 | rs1480845726 | 1:161193990 | G/A | GG/GA/AA | 498 | A/V | 0 |  | 199/1/0 | 196/4/0 | 1.77E-01 |
| SNP145 | rs1318958775 | 1:161193982 | A/G | AA/AG/GG | 501 | C/R | 0 |  | 198/2/0 | 194/6/0 | 1.53E-01 |
| SNP146 | rs1189794230 | 1:161193976 | C/A | CC/CA/AA | 503 | G/C | 0.03 |  | 157/43/0 | 156/40/4 | 9.04E-01 |
| SNP147 | rs748963697 | 1:161193970 | G/A | GG/GA/AA | 505 | R/C | 0.05 |  | 178/22/0 | 188/12/0 | 7.30E-02 |
| SNP148 | rs141276844 | 1:161193964 | G/A | GG/GA/AA | 507 | L/F | 0 |  | 200/0/0 | 193/7/0 | 7.60E-03 |
| SNP149 | rs1456113091 | 1:161193961 | G/A | GG/GA/AA | 508 | H/Y | 0.03 |  | 168/30/2 | 181/16/3 | 5.13E-02 |
| SNP150 | rs911869982 | 1:161193816 | G/A | GG/GA/AA | 520 | A/V | 0.04 |  | 179/18/3 | 187/13/0 | 1.51E-01 |
| SNP151 | rs1387690032 | 1:161193813 | C/G | CC/CG/GG | 521 | G/A | 0.02 |  | 189/11/0 | 194/6/0 | 2.15E-01 |
| SNP152 | rs1485965919 | 1:161193804 | C/A | CC/CA/AA | 524 | G/V | 0 |  | 170/26/4 | 133/59/8 | 1.59E-05 |
| SNP153 | rs751931596 | 1:161193788 | C/G | CC/CG/GG | 529 | W/C | 0 |  | 199/1/0 | 187/12/1 | 1.10E-03 |
| SNP154 | rs1321212264 | 1:161193787 | C/T | CC/CT/TT | 530 | G/S | 0.01 |  | 182/17/1 | 180/16/4 | 7.33E-01 |
| SNP155 | rs776156678 | 1:161193777 | G/C | GG/GC/CC | 533 | S/C | 0 |  | 168/32/0 | 166/34/0 | 7.88E-01 |
| SNP156 | rs761421146 | 1:161193778 | A/G | AA/AG/GG | 533 | S/P | 0 |  | 200/0/0 | 192/8/0 | 7.60E-03 |
| SNP157 | rs768311173 | 1:161193774 | C/T | CC/CT/TT | 534 | R/Q | 0 |  | 183/17/0 | 184/16/0 | 3.53E-02 |
| SNP158 | rs1294691077 | 1:161193769 | A/G | AA/AG/GG | 536 | C/R | 0 |  | 158/42/0 | 151/45/4 | 4.04E-01 |
| SNP159 | rs772202883 | 1:161193759 | C/T | CC/CT/TT | 539 | G/D | 0 |  | 166/33/1 | 165/35/0 | 8.95E-01 |
| SNP160 | rs1206776400 | 1:161193741 | C/T | CC/CT/TT | 545 | R/Q | 0 |  | 193/7/0 | 183/17/0 | 3.53E-02 |
| SNP161 | rs1164707137 | 1:161193739 | C/T | CC/CT/TT | 546 | D/N | 0.01 |  | 138/59/3 | 148/44/8 | 1.41E-02 |
| SNP162 | rs1370490084 | 1:161193736 | A/C | AA/AC/CC | 547 | C/G | 0 |  | 196/4/0 | 189/11/0 | 6.54E-02 |
| SNP163 | rs17855815 | 1:161193721 | G/T | GG/GT/TT | 552 | P/T | 0 |  | 189/11/0 | 184/16/0 | 3.19E-01 |
| SNP164 | rs755517863 | 1:161193718 | G/A | GG/GA/AA | 553 | R/W | 0 |  | 158/41/1 | 150/44/6 | 3.42E-01 |
| SNP165 | rs79052081 | 1:161193693 | C/G | CC/CG/GG | 561 | G/A | 0 |  | 197/3/0 | 193/7/0 | 2.00E-01 |
| SNP166 | rs35674362 | 1:161193691 | G/A | GG/GA/AA | 562 | R/C | 0.01 |  | 173/24/3 | 158/39/3 | 4.71E-02 |
| SNP167 | rs142207487 | 1:161193687 | C/T | CC/CT/TT | 563 | R/H | 0.02 |  | 169/30/1 | 145/48/7 | 3.49E-03 |
| SNP168 | rs1378022599 | 1:161193684 | G/C | GG/GC/CC | 564 | T/S | 0.01 |  | 159/40/1 | 174/25/1 | 4.46E-02 |
| SNP169 | rs759418589 | 1:161193681 | C/T | CC/CT/TT | 565 | R/H | 0 |  | 193/7/0 | 194/6/0 | 7.78E-01 |
| SNP170 | rs147127522 | 1:161193676 | G/A | GG/GA/AA | 567 | R/C | 0.01 |  | 177/23/0 | 170/30/0 | 3.02E-01 |
| SNP171 | rs773407656 | 1:161193672 | G/A | GG/GA/AA | 568 | S/F | 0 |  | 186/12/2 | 158/34/8 | 5.47E-05 |
| SNP172 | rs1272676091 | 1:161193669 | C/G | CC/CG/GG | 569 | C/S | 0 |  | 194/6/0 | 190/10/0 | 3.07E-01 |
| SNP173 | rs1410282522 | 1:161193388 | G/A | GG/GA/AA | 579 | A/V | 0.01 |  | 174/23/3 | 167/32/1 | 3.24E-01 |
| SNP174 | rs1233032576 | 1:161193640 | C/T | CC/CT/TT | 579 | A/T | 0.01 |  | 169/28/3 | 144/49/7 | 2.45E-03 |
| SNP175 | rs1480876833 | 1:161193385 | A/G | AA/AG/GG | 580 | L/P | 0.02 |  | 189/11/0 | 191/9/0 | 6.46E-01 |
| SNP176 | rs200258484 | 1:161193376 | C/T | CC/CT/TT | 583 | R/H | 0 |  | 176/22/2 | 168/30/2 | 2.49E-01 |
| SNP177 | rs768620886 | 1:161193377 | G/A | GG/GA/AA | 583 | R/C | 0 |  | 169/30/1 | 187/11/2 | 4.02E-03 |
| SNP178 | rs746213424 | 1:161193374 | C/T | CC/CT/TT | 584 | E/K | 0.01 |  | 192/8/0 | 191/9/0 | 8.04E-01 |
| SNP179 | rs752405036 | 1:161193353 | T/G | TT/TG/GG | 591 | N/H | 0.01 |  | 178/22/0 | 183/16/1 | 3.99E-01 |
| SNP180 | rs368133764 | 1:161193322 | G/A | GG/GA/AA | 601 | P/L | 0.05 |  | 158/39/3 | 146/47/7 | 1.60E-01 |
| SNP181 | rs760642857 | 1:161193313 | A/G | AA/AG/GG | 604 | M/T | 0.01 |  | 184/15/1 | 187/13/0 | 5.63E-01 |
| SNP182 | rs1261718264 | 1:161193306 | C/G | CC/CG/GG | 606 | W/C | 0 |  | 187/13/0 | 179/21/0 | 1.51E-01 |
| SNP183 | rs746411811 | 1:161193301 | G/A | GG/GA/AA | 608 | P/L | 0 |  | 169/27/4 | 172/21/7 | 6.72E-01 |
| SNP184 | rs148961956 | 1:161193298 | C/T | CC/CT/TT | 609 | R/H | 0 |  | 195/5/0 | 189/11/0 | 1.26E-01 |
| SNP185 | rs779357556 | 1:161193299 | G/A | GG/GA/AA | 609 | R/C | 0 |  | 185/15/0 | 187/13/0 | 6.95E-01 |
| SNP186 | rs749731245 | 1:161193296 | A/C | AA/AC/CC | 610 | Y/D | 0 |  | 158/37/5 | 154/35/11 | 6.29E-01 |
| SNP187 | rs1294599008 | 1:161193292 | G/A | GG/GA/AA | 611 | T/I | 0 |  | 196/4/0 | 188/12/0 | 4.12E-02 |
| SNP188 | rs751007880 | 1:161193287 | C/T | CC/CT/TT | 613 | V/M | 0 |  | 189/11/0 | 193/7/0 | 3.35E-01 |
| SNP189 | rs1421158630 | 1:161193281 | G/T | GG/GT/TT | 615 | P/T | 0.01 |  | 170/27/3 | 171/21/8 | 8.88E-01 |
| SNP190 | rs758291395 | 1:161193275 | C/T | CC/CT/TT | 617 | D/N | 0.01 |  | 165/35/0 | 176/24/0 | 1.21E-01 |
| SNP191 | rs1224438823 | 1:161193270 | C/G | CC/CG/GG | 618 | Q/H | 0 |  | 198/2/0 | 187/13/0 | 3.79E-03 |
| SNP192 | rs767653927 | 1:161193269 | A/C | AA/AC/CC | 619 | C/G | 0 |  | 191/9/0 | 192/8/0 | 8.04E-01 |
| SNP193 | rs371416738 | 1:161193256 | C/A | CC/CA/AA | 623 | C/F | 0 |  | 158/40/2 | 143/46/11 | 8.22E-02 |
| SNP194 | rs1319376302 | 1:161193245 | C/T | CC/CT/TT | 627 | A/T | 0 |  | 181/19/0 | 183/17/0 | 7.27E-01 |
| SNP195 | rs753708382 | 1:161193238 | C/T | CC/CT/TT | 629 | G/D | 0 |  | 187/13/0 | 196/4/0 | 2.57E-02 |
| SNP196 | rs1392836209 | 1:161193230 | A/T | AA/AT/TT | 632 | Y/N | 0.04 |  | 176/23/1 | 167/28/5 | 1.98E-01 |
| SNP197 | rs1000756366 | 1:161193223 | A/T | AA/AT/TT | 634 | L/Q | 0 |  | 154/46/0 | 163/37/0 | 2.67E-01 |
| SNP198 | rs143851585 | 1:161193214 | C/T | CC/CT/TT | 637 | R/Q | 0.03 |  | 158/42/0 | 174/22/4 | 3.32E-02 |
| SNP199 | rs763981957 | 1:161192239 | A/G | AA/AG/GG | 638 | V/A | 0 |  | 181/19/0 | 167/23/10 | 3.74E-02 |
| SNP200 | rs753529783 | 1:161192240 | C/G | CC/CG/GG | 638 | V/L | 0.01 |  | 192/8/0 | 182/13/5 | 4.25E-02 |
| SNP201 | rs1406629683 | 1:161192221 | C/G | CC/CG/GG | 644 | C/S | 0 |  | 170/30/0 | 171/29 | 8.88E-01 |
| SNP202 | rs925057053 | 1:161192215 | G/A | GG/GA/AA | 646 | P/L | 0.03 |  | 155/45/0 | 162/37/1 | 3.88E-01 |
| SNP203 | rs1210373801 | 1:161192216 | G/A | GG/GA/AA | 646 | P/S | 0.01 |  | 154/45/1 | 163/34/3 | 2.67E-01 |
| SNP204 | rs977710745 | 1:161192206 | G/C | GG/GC/CC | 649 | S/C | 0.01 |  | 165/34/1 | 170/28/2 | 4.98E-01 |
| SNP205 | rs767912629 | 1:161192203 | G/A | GG/GA/AA | 650 | S/L | 0 |  | 159/38/3 | 174/23/3 | 4.46E-02 |
| SNP206 | rs774609955 | 1:161192188 | C/T | CC/CT/TT | 655 | G/D | 0 |  | 193/7/0 | 187/13/0 | 1.69E-01 |
| SNP207 | rs1358979298 | 1:161192183 | A/T | AA/AT/TT | 657 | C/S | 0 |  | 199/1/0 | 188/12/0 | 1.92E-03 |
| SNP208 | rs1480911158 | 1:161192180 | T/G | TT/TG/GG | 658 | I/L | 0.01 |  | 171/29/0 | 178/22/0 | 2.94E-01 |
| SNP209 | rs570236275 | 1:161192162 | G/A | GG/GA/AA | 664 | R/C | 0.01 |  | 187/13/0 | 174/26/0 | 2.84E-02 |
| SNP210 | rs745360883 | 1:161192155 | A/G | AA/AG/GG | 666 | I/T | 0 |  | 197/3/0 | 192/8/0 | 1.26E-01 |
| SNP211 | rs116361265 | 1:161192143 | T/G | TT/TG/GG | 670 | K/T | 0.01 |  | 182/18/0 | 193/7/0 | 2.31E-02 |
| SNP212 | rs749255932 | 1:161192133 | G/C | GG/GC/CC | 673 | D/E | 0 |  | 159/40/1 | 169/30/1 | 1.93E-01 |
| SNP213 | rs376630100 | 1:161192126 | T/C | TT/TC/CC | 676 | M/V | 0.02 |  | 187/13/0 | 184/16/0 | 5.63E-01 |
| SNP214 | rs767813779 | 1:161192117 | C/T | CC/CT/TT | 679 | G/R | 0.01 |  | 192/8/0 | 189/11/0 | 4.81E-01 |
| SNP215 | rs3134667 | 1:161192108 | C/T | CC/CT/TT | 682 | G/S | 0.04 |  | 172/27/1 | 163/31/6 | 2.23E-01 |
| SNP216 | rs374106325 | 1:161192068 | A/T | AA/AT/TT | 695 | F/Y | 0.04 |  | 170/24/6 | 185/15/0 | 1.76E-02 |
| SNP217 | rs762249128 | 1:161191560 | C/T | CC/CT/TT | 698 | G/R | 0 |  | 189/11/0 | 169/30/1 | 1.11E-03 |
| SNP218 | rs754163569 | 1:161191557 | A/G | AA/AG/GG | 699 | Y/H | 0 |  | 171/24/5 | 183/16/1 | 6.00E-02 |
| SNP219 | rs773982106 | 1:161191547 | A/G | AA/AG/GG | 702 | V/A | 0 |  | 199/1/0 | 188/12/0 | 1.92E-03 |
| SNP220 | rs760202403 | 1:161191545 | C/A | CC/CA/AA | 703 | V/F | 0 |  | 195/5/0 | 190/10/0 | 1.88E-01 |
| SNP221 | rs1159271642 | 1:161191538 | A/G | AA/AG/GG | 705 | I/T | 0 |  | 159/37/4 | 168/40/2 | 2.44E-01 |
| SNP222 | rs1162998745 | 1:161191539 | T/C | TT/TC/CC | 705 | I/V | 0.01 |  | 196/4/0 | 189/11/0 | 6.54E-02 |
| SNP223 | rs748124639 | 1:161191536 | G/A | GG/GA/AA | 706 | P/S | 0 |  | 197/3/0 | 190/10/0 | 4.84E-02 |
| SNP224 | rs903703821 | 1:161191529 | C/T | CC/CT/TT | 708 | G/E | 0 |  | 170/30/0 | 171/28/1 | 8.88E-01 |
| SNP225 | rs745913279 | 1:161191508 | C/T | CC/CT/TT | 715 | R/Q | 0 |  | 199/1/0 | 191/9/0 | 1.04E-02 |
| SNP226 | rs113816084 | 1:161191509 | G/A | GG/GA/AA | 715 | R/W | 0 |  | 191/9/0 | 193/7/0 | 6.10E-01 |
| SNP227 | rs778890470 | 1:161191504 | C/A | CC/CA/AA | 716 | Q/H | 0.02 |  | 179/20/1 | 158/39/3 | 3.95E-03 |
| SNP228 | rs757718427 | 1:161191499 | C/T | CC/CT/TT | 718 | G/E | 0.03 |  | 159/41/0 | 162/35/3 | 7.06E-01 |
| SNP229 | rs1256912569 | 1:161191477 | G/C | GG/GC/CC | 725 | I/M | 0.02 |  | 186/14/0 | 177/23/0 | 1.20E-01 |
| SNP230 | rs775171599 | 1:161191445 | T/C | TT/TC/CC | 736 | Y/C | 0 |  | 185/13/2 | 167/27/6 | 5.61E-03 |
| SNP231 | rs1397348423 | 1:161191440 | G/C | GG/GC/CC | 738 | L/V | 0 |  | 172/23/5 | 164/28/8 | 2.75E-01 |
| SNP232 | rs746084090 | 1:161191424 | G/A | GG/GA/AA | 743 | T/M | 0.02 |  | 168/28/4 | 184/13/3 | 1.38E-02 |
| SNP233 | rs1289448605 | 1:161191421 | A/G | AA/AG/GG | 744 | L/P | 0 |  | 173/22/5 | 186/13/1 | 3.21E-02 |
| SNP234 | rs1159179998 | 1:161191410 | G/A | GG/GA/AA | 748 | P/S | 0.03 |  | 172/24/4 | 169/28/3 | 6.72E-01 |
| SNP235 | rs757273170 | 1:161191401 | C/T | CC/CT/TT | 751 | V/M | 0 |  | 175/25/0 | 171/28/1 | 5.58E-01 |
| SNP236 | rs1359805958 | 1:161191394 | A/G | AA/AG/GG | 753 | L/P | 0 |  | 186/14/0 | 173/27/0 | 3.21E-02 |
| SNP237 | rs1484891983 | 1:161191385 | G/A | GG/GA/AA | 756 | A/V | 0.04 |  | 185/14/1 | 176/22/2 | 1.29E-01 |
| SNP238 | rs139491371 | 1:161191373 | C/T | CC/CT/TT | 760 | R/H | 0.03 |  | 160/38/2 | 162/36/2 | 8.01E-01 |
| SNP239 | rs376187908 | 1:161191364 | C/A | CC/CA/AA | 763 | G/V | 0 |  | 187/13/0 | 182/18/0 | 3.50E-01 |
| SNP240 | rs1311533880 | 1:161191365 | C/A | CC/CA/AA | 763 | G/W | 0 |  | 197/3/0 | 185/15/0 | 3.80E-03 |
| SNP241 | rs761401967 | 1:161191347 | C/G | CC/CG/GG | 769 | E/Q | 0 |  | 172/20/8 | 153/28/19 | 1.49E-02 |
| SNP242 | rs1369336339 | 1:161191325 | G/A | GG/GA/AA | 776 | P/L | 0 |  | 170/27/3 | 182/18/0 | 6.48E-02 |
| SNP243 | rs201242393 | 1:161191307 | G/T | GG/GT/TT | 782 | T/K | 0 |  | 172/23/5 | 158/38/4 | 6.54E-02 |
| SNP244 | rs745735551 | 1:161191296 | G/C | GG/GC/CC | 786 | L/V | 0 |  | 193/7/0 | 181/19/0 | 1.49E-02 |
| SNP245 | rs781559105 | 1:161191269 | G/A | GG/GA/AA | 795 | R/C | 0 |  | 160/40/0 | 157/43/0 | 7.11E-01 |
| SNP246 | rs1403805603 | 1:161191260 | A/G | AA/AG/GG | 798 | Y/H | 0 |  | 196/4/0 | 186/14/0 | 1.59E-02 |
| SNP247 | rs760634103 | 1:161191256 | C/T | CC/CT/TT | 799 | S/N | 0 |  | 187/13/0 | 183/16/1 | 4.48E-01 |
| SNP248 | rs114152196 | 1:161191254 | A/C | AA/AC/CC | 800 | F/V | 0 |  | 172/21/7 | 152/31/17 | 1.08E-02 |
| SNP249 | rs767560823 | 1:161191251 | A/G | AA/AG/GG | 801 | F/L | 0 |  | 185/15/0 | 189/9/2 | 4.17E-01 |
| SNP250 | rs1240936333 | 1:161191244 | G/A | GG/GA/AA | 803 | P/L | 0 |  | 174/26/0 | 188/12/0 | 1.70E-02 |
| SNP251 | rs527950908 | 1:161191241 | C/T | CC/CT/TT | 804 | R/Q | 0 |  | 189/10/1 | 178/21/1 | 4.56E-02 |
| SNP252 | rs753559369 | 1:161191221 | G/A | GG/GA/AA | 811 | R/C | 0.05 |  | 160/39/1 | 184/15/1 | 5.44E-04 |
| SNP253 | rs1191247835 | 1:161191188 | C/T | CC/CT/TT | 822 | A/T | 0.04 |  | 177/23/0 | 184/13/3 | 2.38E-01 |
| SNP254 | rs759910530 | 1:161191183 | C/G | CC/CG/GG | 823 | Q/H | 0.02 |  | 183/15/2 | 178/22/0 | 3.99E-01 |
| SNP255 | rs773407497 | 1:161191171 | G/C | GG/GC/CC | 827 | I/M | 0.02 |  | 167/33/0 | 172/24/4 | 4.87E-01 |
| SNP256 | rs927775299 | 1:161191167 | G/A | GG/GA/AA | 829 | R/W | 0 |  | 174/25/1 | 188/10/2 | 1.70E-02 |
| SNP257 | rs114728887 | 1:161191164 | G/A | GG/GA/AA | 830 | R/W | 0 |  | 160/37/3 | 182/16/2 | 1.78E-03 |
| SNP258 | rs1336605919 | 1:161191149 | C/T | CC/CT/TT | 835 | G/S | 0.04 |  | 173/24/3 | 169/30/1 | 5.70E-01 |

^a^ X/Y represents wild type allele/variant allele.

^b^ XX/XY/YY represents wild type homozygote/heterozygote/variant type homozygote.

^c^ N_XX_/N_XY_/N_YY_ = the number of subjects with XX genotype/ the number of subjects with XY genotype / the number of subjects with YY genotype.

**Supplementary Table S4 The interactive values of ADAMTS4 SNPs and causes (including HBV and AFB1) for hepatocellular carcinoma risk**

| Interactive variable | OR_interaction_ | 95% CI_interaction_ | *P*_trend_ |
| --- | --- | --- | --- |
| Positive-HBV × rs538321148-TT | 7.30 | 2.02-26.35 | 2.43 × 10^-3^ |
| Positive-HBV × rs1014509103-AA | 3.96 | 1.41-11.13 | 9.08 × 10^-3^ |
| Positive-AFB1 × rs538321148-CT | 3.77 | 2.45-5.81 | 1.80 × 10^-9^ |
| Positive-AFB1 × rs538321148-TT | 3.93 | 1.65-9.34 | 1.95 × 10^-3^ |
| Positive-AFB1 × rs1014509103-GA | 1.72 | 1.06-2.81 | 0.03 |
| Positive-AFB1 × rs1014509103-AA | 12.72 | 4.72-34.49 | 5.30 × 10^-7^ |
